# Complementary ecosystem services from multiple land uses highlight the importance of tropical mosaic landscapes

**DOI:** 10.1007/s13280-023-01888-3

**Published:** 2023-06-07

**Authors:** Estelle Raveloaritiana, Annemarie Wurz, Kristina Osen, Marie Rolande Soazafy, Ingo Grass, Dominic Andreas Martin, Claudine Bemamy, Hery Lisy Tiana Ranarijaona, Cortni Borgerson, Holger Kreft, Dirk Hölscher, Bakolimalala Rakouth, Teja Tscharntke

**Affiliations:** 1 Plant Biology and Ecology Department, University of Antananarivo, Antananarivo, Madagascar; 2 Agroecology, Department of Crop Sciences, University of Goettingen, Göttingen, Germany; 3 Sustainable Agricultural Systems and Engineering Laboratory, School of Engineering, Westlake University, Hangzhou, China; 4 Conservation Ecology, Department of Biology, Philipps-Universität Marburg, Marburg, Germany; 5 Tropical Silviculture and Forest Ecology, University of Goettingen, Göttingen, Germany; 6 Natural and Environmental Sciences, Regional University Centre of the SAVA Region (CURSA), Antalaha, Madagascar; 7 Natural Ecosystems (EDEN), University of Mahajanga, Mahajanga, Madagascar; 8 Ecology of Tropical Agricultural Systems, University of Hohenheim, Stuttgart, Germany; 9 Biodiversity, Macroecology and Biogeography, University of Goettingen, Göttingen, Germany; 10 Department of Geography, University of Zurich, Zurich, Switzerland; 11 Diversity Turn in Land Use Sciences Research Project, Sambava, Madagascar; 12 Department of Anthropology, Montclair State University, Montclair, USA; 13 Centre for Biodiversity and Sustainable Land Use (CBL), University of Goettingen, Göttingen, Germany

**Keywords:** Biodiversity conservation, Fallow lands, Plant use, Rural livelihoods, Socio-ecological trade-offs, Vanilla agroforestry

## Abstract

Tropical agricultural landscapes often consist of a mosaic of different land uses, yet little is known about the spectrum of ecosystem service bundles and materials they provide to rural households. We interviewed 320 households on the different benefits received from prevalent land-use types in north-eastern Madagascar (old-growth forests, forest fragments, vanilla agroforests, woody fallows, herbaceous fallows, and rice paddies) in terms of ecosystem services and plant uses. Old-growth forests and forest fragments were reported as important for regulating services (e.g. water regulation), whilst fallow lands and vanilla agroforests as important for provisioning services (food, medicine, fodder). Households reported the usage of 285 plant species (56% non-endemics) and collected plants from woody fallows for varying purposes, whilst plants from forest fragments, predominantly endemics, were used for construction and weaving. Multiple land-use types are thus complementary for providing ecosystem services, with fallow lands being particularly important. Hence, balancing societal needs and conservation goals should be based on diversified and comprehensive land management.

**Supplementary Information:**

The online version contains supplementary material available at 10.1007/s13280-023-01888-3.

## Introduction

Ecosystem services, such as products from the natural environment, support rural livelihoods in many tropical countries (Sunderlin et al. 2005; Angelsen et al. 2014). These ecosystem services are often derived from specific ecosystems, for example, forests, and are particularly important for poor rural households (Angelsen et al. 2014; Wunder et al. 2014). However, many tropical forests in sub-Saharan Africa have been transformed into agricultural lands (Curtis et al. 2018). For instance, more than half of new agricultural lands in sub-Saharan Africa have been derived from natural forests during the late twentieth century (Gibbs et al. 2010), primarily through shifting cultivation (Ickowitz 2006). In case of shifting cultivation, forests or woodlands are cleared and burned to plant crops for one to three years, after which the land is left as fallow (i.e. unplanted) for a period of five to 30 or more years (Thrupp et al. 1997).

In tropical regions, shifting cultivation, combined with other agricultural techniques, has led to mosaic landscapes comprising forests, small-scale agriculture, and fallow lands (Finegan and Nasi 2004). These land-use changes have led to complex sets of ecosystem service provision within tropical agricultural landscapes (Clough et al. 2016). For instance, tropical agroforestry systems can provide valuable ecosystem services beyond crop production, including carbon storage and pollination (Tscharntke et al. 2011; De Beenhouwer et al. 2013). Agroforests can also provide a source of income for households and contribute to the multifunctionality of mosaic landscapes (Plieninger et al. 2020). For fallow lands, forming part of shifting cultivation systems, several studies have reported their importance in terms of the provision of diverse products to rural households in many regions, particularly in sub-Saharan Africa (e.g. Ambrose-Oji 2003; Pouliot and Treue 2013; Zaehringer et al. 2017). Yet, fallow lands are often undervalued compared to forests and agroforests in terms of ecosystem services beyond soil regeneration (De Beenhouwer et al. 2013; Peña Valderrama 2020), as shifting cultivation is associated with numerous socio-ecological impacts (Thrupp et al. 1997). Whilst all land-use systems may provide ecosystem services, their realised importance depends on people perceiving and using these lands for such services (Daw et al. 2011; Sayer et al. 2013; IPBES 2022). Importantly, the importance may differ along various socio-economic and demographic axes (Quintas-Soriano et al. 2018), highlighting the need to account for this diversity in research to provide a meaningful recommendation for sustainable land management strategies.

On top of ecosystem services, research for sustainable land-use solutions should also take into account biodiversity to develop large-scale land management strategies that benefit both people and nature (Kremen and Merenlender 2018). Socio-ecological research must, therefore, consider the material needs of rural households (e.g. medicine, food, building material, and energy) to develop effective land management strategies (Smith and Sullivan 2014; Wanger et al. 2020). Here, we focus on rural households’ perceptions of ecosystem services provided by prevalent land-use types in Madagascar, a global biodiversity hotspot (Myers et al. 2000). Malagasy rural households highly depend on the availability of natural resources, especially forest-derived resources, for their livelihoods (Scales 2014a), yet the country has lost 44% of its forest cover over the last six decades (Vieilledent et al. 2018).

In the north-eastern part of Madagascar, most old-growth forests have been transformed to shifting cultivation for hillside rice production and agroforests for cash and subsistence crops (Curtis et al. 2018; Llopis et al. 2019). In those agroforests, vanilla is the main cash crop (Hänke et al. 2018), as the region is the world’s largest producer of vanilla with roughly 40% global market share (The World Bank 2019). Consequently, the landscape comprises currently forest fragments, small-scale vanilla agroforests, rice paddies, and lands for subsistence farming at various stages of the shifting cultivation cycle (Zaehringer et al. 2016; Martin et al. 2022). In terms of biodiversity conservation, forests and agroforests have a much higher value in maintaining endemic plant diversity than fallow lands (Osen et al. 2021; Raveloaritiana et al. 2021). However, these prevalent land-use types have so far not been comprehensively compared within the spectrum of perceived ecosystem service bundles and materials they provide to rural households; but such analyses are needed to identify trade-offs associated with land-use change (de Groot et al. 2010). Such comprehensive trade-off analyses are rarely done, but highly important for a pluralistic perspective of mosaic landscapes to generate tangible recommendations for sustainable land management strategies that take into account local context and societal as well as biodiversity conservation needs (Pascual et al. 2021; IPBES 2022). Here, biodiversity studies can be integrated into ecosystem service assessments to inform policies that benefit plant conservation as well as human well-being.

In this study, we investigate the importance of forests and different agricultural land-use types, including agroforests, fallow lands, and rice paddies, for rural households’ livelihoods in Madagascar with a focus on ecosystem services and plant uses. This research aims to answer: (1) How important are the prevalent land-use types in ensuring provisioning, supporting, regulating, and cultural ecosystem services, based on the benefits perceived by rural households? (2) Which land-use types are used to collect materials for rural household provision (plants for medicine, food, construction, firewood, charcoal, fodder, and weaving)? (3) Which and how many plant species are collected from each land-use type? (4) How do the perceived benefits, harvested materials, and plant species change across land-use types?

## Materials and methods

### Study area and village selection

We conducted this study in the SAVA region, in north-eastern Madagascar (Figure S1), which has a humid climate and a climax vegetation characterised by tropical rainforests (Moat and Smith 2007). The SAVA region still retains a large proportion of forest cover compared to most regions in Madagascar (Vieilledent et al. 2018). Hence, this region is not only important for biodiversity conservation but also for maintaining ecosystem services (Rogers et al. 2010). Moreover, the agricultural landscape mosaics in the region are dominated by rice and vanilla agroforests, combined with forest fragments and fallow lands forming part of a shifting cultivation system (Zaehringer et al. 2016; Hänke et al. 2018; Martin et al. 2022). This land-use diversity makes the region an interesting area for the study of ecosystem services and conservation. For our study, we selected 10 villages (Figure S1) from the 60 villages of a baseline study conducted in the region by Hänke et al. (2018, see SI Section 1 for more details).

### Land-use types classification and conceptual framework

We considered the six most prevalent land-use types in the region: old-growth forests, forest fragments, vanilla agroforests, woody fallows, herbaceous fallows, and rice paddies (Hänke et al. 2018; Martin et al. 2022)*.* We defined old-growth forests (Malagasy: *ala mikitroka*) as the least disturbed forests, which nowadays only occur within protected areas (Schüßler et al. 2020). We characterised forest fragments (Malagasy: *ala mitsitokotoko*) as the remaining forests outside of protected areas, which are usually heavily used by people. We defined vanilla agroforests (Malagasy: *lavanio*) as croplands in which vanilla orchids are farmed on support trees under shade trees; woody fallows (Malagasy: *savoka*) as lands forming part of shifting cultivation cycles dominated by trees and/or woody plants; herbaceous fallows (Malagasy: *matrangy*) as lands forming part of shifting cultivation cycles dominated by herbaceous vegetation; and rice paddies (Malagasy: *oraka*) as irrigated paddy rice fields.

In our study, we explore the complex relationship between people and nature with a focus on the land-use types described above and rural livelihoods based on perceptions. For this, we adopted the framework by Haines-Young and Potschin (2010), which links biodiversity, ecosystem services, and human well-being. First, we looked into the broad types of ecosystem services (i.e. regulating, supporting, provisioning, and cultural services) which rural households perceive to obtain from various land-use types. Second, we specifically focus on provisioning services that contribute highly to livelihoods via basic materials for everyday needs (e.g. shelter or construction and weaving, food, fodder, medicine, and energy or firewood and charcoal; Millennium Ecosystem Assessment 2005). Third, to have a direct link with biodiversity conservation, we focused on plant species used for food, medicine, construction, firewood, charcoal, fodder, and weaving, as north-eastern Madagascar is a global centre of vascular plant diversity (Mutke et al. 2011).

### Selection of households, survey structure, and research ethics

We randomly selected 32 households per village from a list of people provided by the leader of each village (e.g. chef de fokontany). Selected households who were not available or did not want to participate in the interview were replaced by other households, which were also randomly chosen from the list. In total, we interviewed 320 households comprising 1567 individuals in 10 villages within the SAVA region.

Whilst the questionnaire of this survey was developed at the University of Antananarivo, which does not have a research ethics committee for social science work, we followed human subjects’ protocol and all participants provided free and informed consent before the start of the study. Each interviewee was also given an option to halt the interview at any time and to withdraw their consent.

We interviewed the head of each household individually for one to two hours depending on the land-use types that the household owned or had access to, as well as the plant species used. We used the Malagasy dialect Betsimisaraka to conduct the interview and we then translated the answers into English afterwards. Prior to the main interview, we asked for basic information about the socio-demographic characteristics of the interviewee, such as age, gender, and educational level. Then, we asked households about (I) their access to, the use of, and their perceived benefits from land-use types and (II) the plants they use for different categories of use and the land-use types they collect them from (Table S1). We asked the questions in the same order and manner for all interviewees throughout all villages.

To understand the overall importance of land-use types for ecosystem services, we first asked whether households had access (by ownership or permission) to each defined land-use type (Table S1) by describing the land-use types. Then, for each land-use type, participants described the benefits (products for use/selling or indirect services) that their households got from that land-use type (excluding the main crop they grow, such as vanilla or rice). Questions about benefits were open response to minimise researcher biases (Neuman 2014), and responses were secondarily categorised into regulating, supporting, provisioning, or cultural services (Millennium Ecosystem Assessment 2005; SI Section 2).

To evaluate the importance of land-use types for collecting plants, we chose seven categories of use for plants: medicine, food, construction, firewood, charcoal, fodder, and weaving (Table S1). These categories of use reflect the predominant use of plants in people’s daily lives. We asked each respondent about the plants they used and the land-use types where plants were collected from, how often (frequency of use), and whether each plant was for household use, selling, or both (Table S1). For the frequency of use, we categorised the answers into three types of frequency: daily to weekly (every day to several times a week), monthly to semi-annually (several times a month to a few times a year), and annually to more rarely (once a year to once a decade or more rarely).

When we did not know the scientific species name of a plant mentioned by the interviewee, we collected herbarium samples (with help of interviewees and local guides) for identification at the Tsimbazaza herbarium (TAN) in Antananarivo, Madagascar. We determined the growth form of each recorded species (tree, liana, shrub, or herb) and the origin (endemic, native non-endemic hereafter called native, or exotic to Madagascar) using two plant databases: Catalogue of the Vascular Plants of Madagascar (Madagascar Catalogue 2019) and the Plants of the World Online (The Royal Botanic Gardens Kew 2019). We excluded 21 local names out of 364 from our dataset for the analysis of the diversity of species used, as we were not able to identify their scientific names due to the absence of specimens and lack of literature linking vernacular to scientific names.

### Data analysis

We conducted all data analysis in R version 4.0.4 (R Core Team 2021). To determine the importance of the mosaic landscapes (i.e. all land-use types combined) in terms of ecosystem services, we calculated the percentage of the households across all villages that reported benefits referring to each type of ecosystem service. To quantify the households that were using plants for each of the seven categories across all villages, we calculated the percentage of households that used at least one plant for each category. To understand the households’ access to land-use types, we counted all households that declared owning or having access to each land-use type across all villages and we visualised the number of households that had specific combinations of land-use types or only one land-use type, using the R-package *ggforce* (Pedersen 2020).

To identify the importance of each land-use type for rural households at village level, we used three different measures at village level: (1) percentage of households benefiting from each ecosystem service type from each land-use type; (2) percentage of households collecting plants for each category of use from each land-use type; and (3) the number of plant species used by households from each land-use type and percentages of each species origin and growth form within each land-use type. Here, the percentage of households was based on the number of households having access to the land-use types instead of the total number of interviewed households per village. The exception to this was regulating services since households can still benefit from this type of ecosystem services without having access to the land-use type. For each measure, we conducted several analyses to determine the importance of each land-use type and to compare each land-use type with one another (Table 1, see SI Section 3 for details).

**Table 1:** Summary of the analyses to determine the importance and the comparisons of the land-use types in terms of ecosystem services and plant use

Measures of importance		Comparison within each land-use type of:	Comparison of all land-use types in terms of:
Ecosystem services	Percentage of household reporting services	Four (4) ecosystem service types Gender of household heads Highest attained educational levels	Each type of ecosystem service
Plant-use categories	Percentage of household reporting use categories	Seven (7) plant-use categories Use frequency groups: daily to weekly, monthly to semi-annually, annually to more rarely Purposes groups: use, selling, use and selling	Each category of plant-use
Plant-use diversity	Number of species used		Number of species used
	Percentage of species origin	Species origin group: endemic, native, or exotic species	
	Percentage of species growth form	Species growth forms: tree, shrub, liana and herb	
	Citation number of species	Harvest location of most cited species	

## Results

On average, 88.4% (SE = ± 2.6) of interviewed households per village reported benefiting from provisioning services from mosaic landscapes, whilst 51.9 ± 6.1% mentioned regulating services. The percentages of households mentioning supporting (7.2 ± 1.5%) and cultural services (6.9 ± 1.9%) were relatively low.

Of provisioning services, high percentages of households in each village collected plants from mosaic landscapes for firewood (95.3 ± 1.4%), medicine (89.7 ± 2.2%), and food (85.3 ± 3.5%, Figure S2). Additionally, 74.1% (± 7.2) of households collected plants for construction, whilst 60.6% (± 6.7) used mosaic landscapes to collect plants for use as fodder for their livestock. Relatively few households reported using plants for charcoal or weaving (11.6 ± 2.7% and 27.5 ± 3.7%, respectively).

### Access to land-use types and their importance for ecosystem service types

**Fig. 1:**
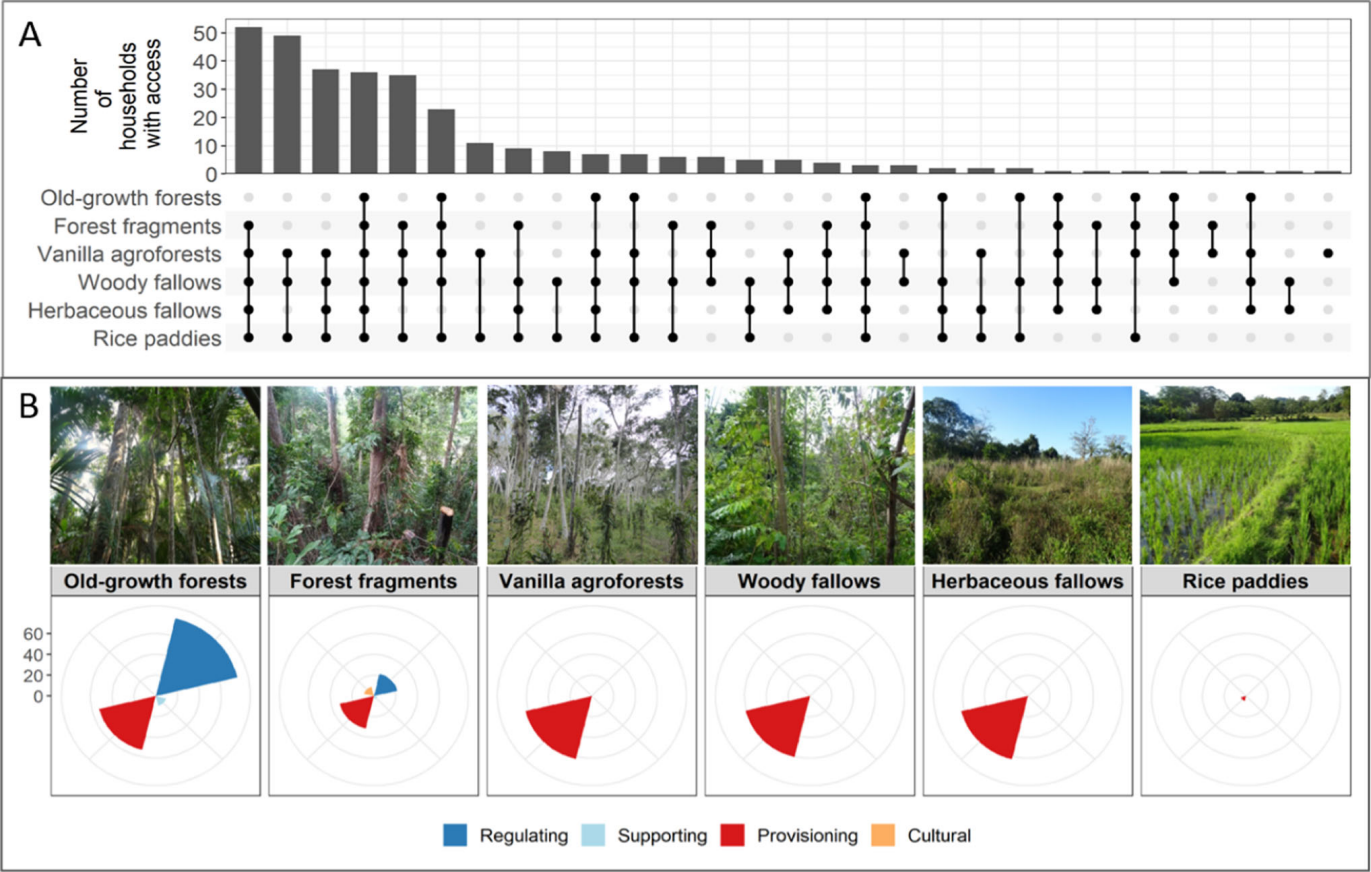
Distribution of access or ownership of land-use types across rural households in north-eastern Madagascar (**A**) and overall perceived importance in terms of each type of ecosystem service excluding crop yields (**B**, see Table S3 for numeric values with standard errors). Vertical bars (**A**) represent the number of households having access to each of the unique combinations of land-use types represented by the connected points. The bars display the number of households with access to the corresponding combination of land-use types (e.g. the combination forest fragments, vanilla agroforests, woody fallows, herbaceous fallows, and rice paddies is accessible to around 50 households). Bars are ordered by the combination of land-use types from highest to lowest accessibility

The land-use types owned or accessed most frequently by households were woody fallows ([mean households’ percentage ± SE] = 95 ± 3.4%), rice paddies (92.2 ± 3.4%), and vanilla agroforest (87.8 ± 4.9%; Table S2). Most households (> 90%) owned or had access to multiple land-use types (Fig. 1A). For the individual importance of land-use types to households, old-growth forests were mostly perceived to deliver regulating services (77 ± 3.6%; primarily for water regulation and air quality; Table S3), provisioning services (53.4 ± 7.5%, principally plants as materials for construction), and supporting services (9.4 ± 1.8%, e.g. animal habitats). Forest fragments were of similar importance as old-growth forests but with the addition of cultural services (9.2 ± 2.8%; e.g. land reserved for descendants; Fig. 1B; Table S3). Vanilla agroforests, woody fallows, and herbaceous fallows were reported only for provisioning services (approximately 60% for each land-use type, Fig. 1B; e.g. firewood and food-related benefits, such as wild food or fruit trees; Table S3).

**Fig. 2:**
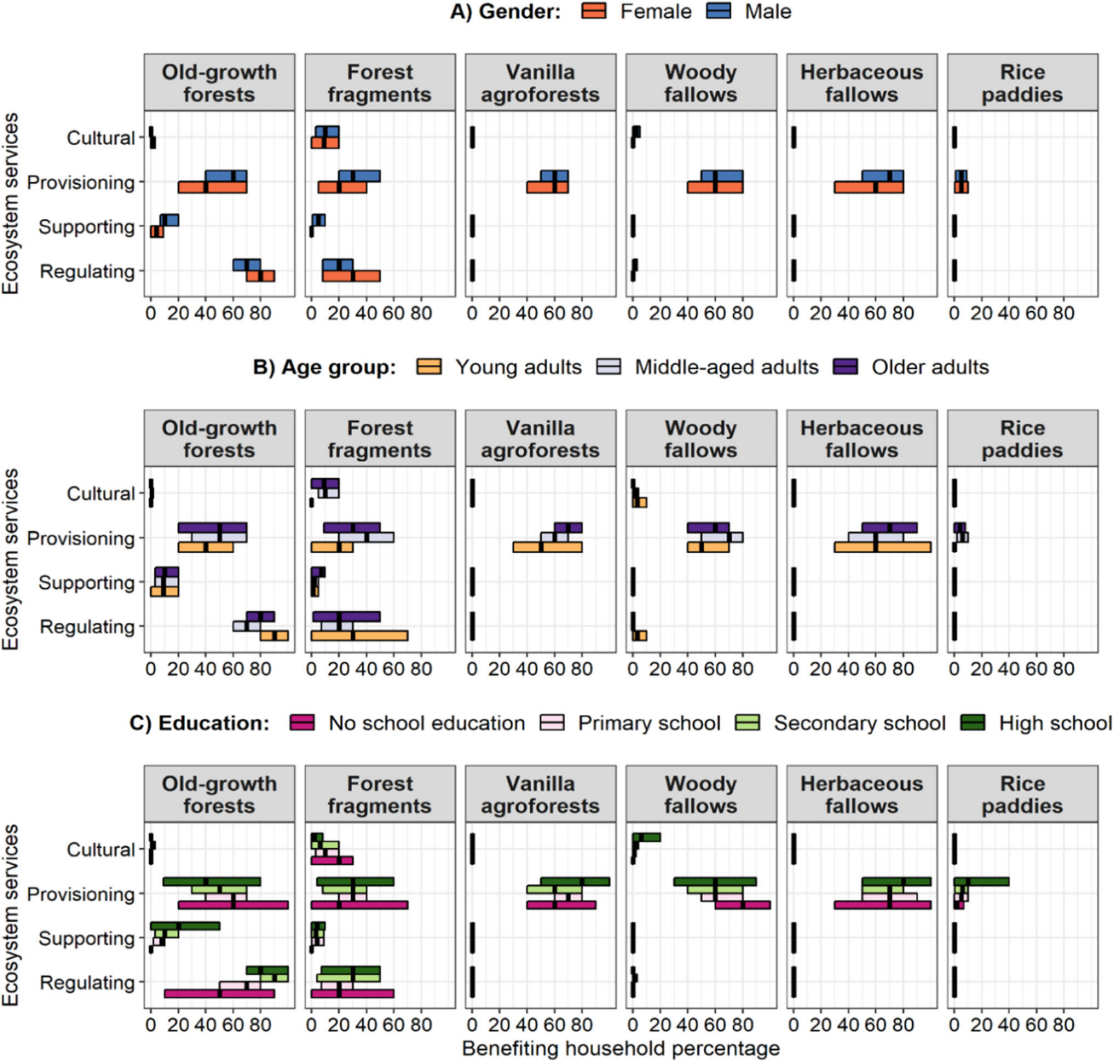
Characteristics of the households perceiving ecosystem services (excluding crop yields) from different land-use types based on gender (**A**), age group (**B**), and education level (**C**) of household heads. The line in the middle of a crossbar represents the mean percentage of households belonging to the group (gender, age group, or education) with access to the land-use type reporting the corresponding benefits, i.e. supporting, regulating, provisioning, or cultural ecosystem services for each land-use type. The limits of the crossbar are the 95% confidence interval of the percentage of the household. For numerical values of mean percentages with SE see Fig. S3

When looking into socio-demographic groups of households’ heads by gender, the percentage of female-headed households reporting regulating services from old-growth forests (80.3 ± 5.9%) was higher than that in male-headed households (73.6 ± 3.9%, Figs. 2A, S3A). Concerning age groups, more young adult household heads perceived regulating services from old-growth forests (92.7 ± 4%) than older (77.7 ± 5.4%) or middle-aged adults household heads (71.5 ± 4.5%, Figs. 2B, S3B). For educational levels, more of the households’ heads that went to secondary or high schools reported regulating services from old-growth forests (secondary school: 88.6 ± 4% and high school: 84.8 ± 8.6%) than less-educated household heads (67 ± 6.3% for primary school, 51.4 ± 15.9% for no school education, Figs. 2C, S3C).

**Fig. 3:**
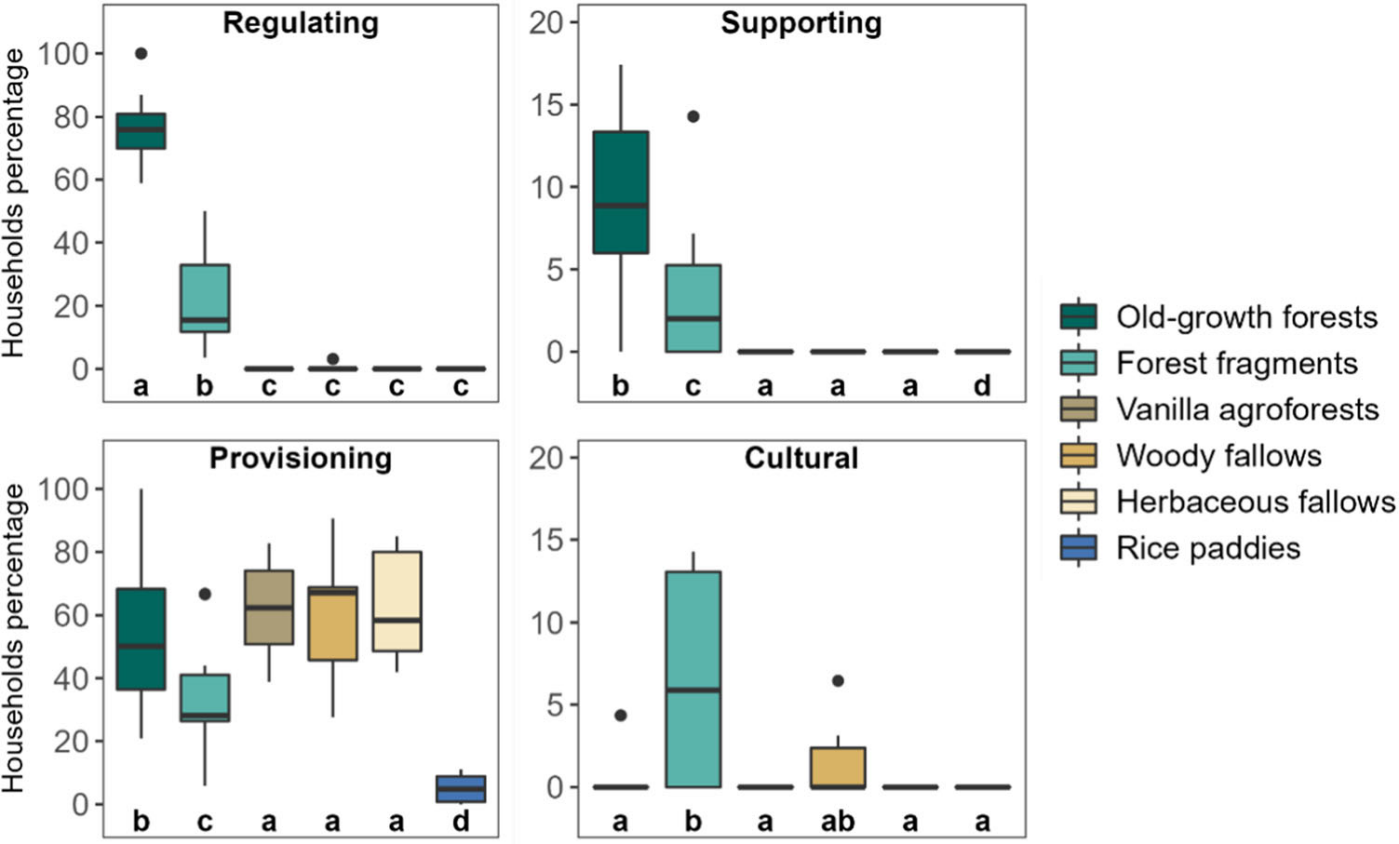
Percentage of households reporting benefits from each type of ecosystem services across land-use types in north-eastern Madagascar. Letters represent the results of post hoc tests with *p* < 0.05 for a significant difference. Lower and upper limits of the box display 25th and 75th percentiles of the observational values, respectively, bold lines are the medians, the lower vertical lines are the 10th percentiles, and the upper vertical lines are 90th percentiles. Note the different scaling of the y-axes

When comparing land-use types for each type of ecosystem service, old-growth forests were the most reported to ensure regulating services ([percentage of households ± SE] = 77 ± 3.6%) and supporting services (9.5 ± 1.8%; Fig. 3; Table S4). Forest fragments were the second most reported land-use type for ensuring regulating (22.1 ± 5.2%) and supporting services (3.5 ± 1.5%), far ahead of other land-use types. For provisioning services, old-growth forests, vanilla agroforests, woody fallows, and herbaceous fallows were the most mentioned land-use types and had a similar percentage of households (53–63%, Table S4), followed by forest fragments. Rice paddies had the lowest percentage of households perceiving benefits from its provisioning services beyond rice production (5 ± 1.4%). For cultural services, households mentioned primarily forest fragments (9.2 ± 2.8%), but the overall value was very low (< 10%, Fig. 3).

### Importance of land-use types for plant use

**Fig. 4:**
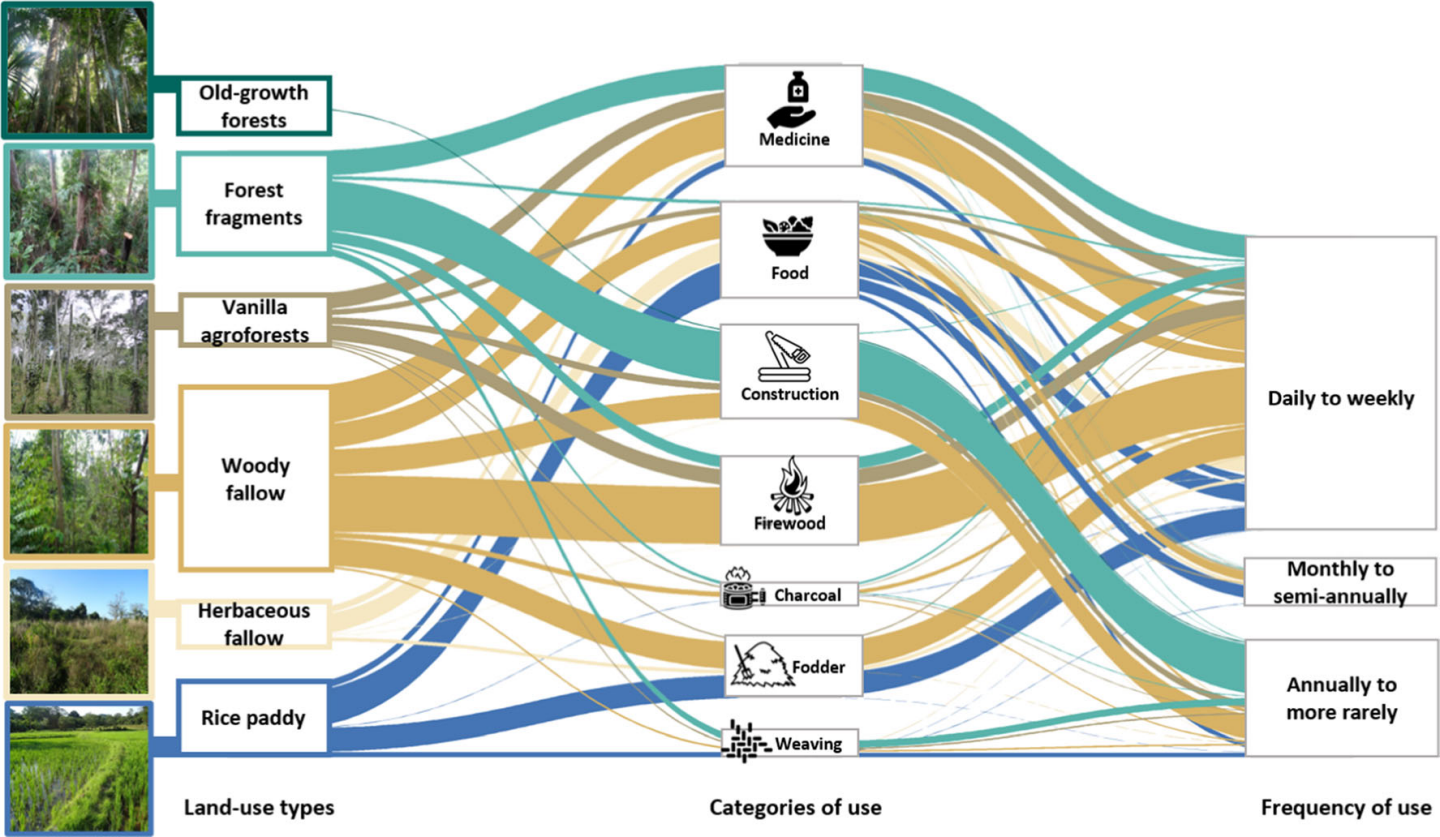
Importance of land-use types to households in north-eastern Madagascar for each category of use of plants and the frequency of use of plants. The width of each link represents the mean of households’ percentage using a corresponding land-use type for each category of use and each type of frequency of use (see Table S5–S6 for numeric results). See reference of the icons in SI Section 2

Across all villages, a high percentage of households reported using forest fragments as a place to collect plants for construction (64.6 ± 5.6%; Table S5, Fig. 4) and medicine (34.2 ± 5.5%). Vanilla agroforests were mainly mentioned for medicinal plants—(20.2 ± 5.2%) and firewood collection (19.2 ± 6.7%) but were rarely used for other categories (Fig. 4; Table S5). Woody fallows were used by a relatively high households’ percentage across all use categories, especially for firewood (74.1 ± 5.5%), medicine (50.7 ± 4.8%), fodder (35.5 ± 8.8%), construction (34.7 ± 6.4%), and food (31.7 ± 6.8%). Herbaceous fallows were used by households as a place to collect plants for food (23.4 ± 4.6%), whilst rice paddies were important for food other than rice, such as leaves from wild plants (46.2 ± 10.5%) and for fodder (31 ± 6.3%). Whilst old-growth forests were only named as a location to collect plants for construction, the percentage of households was very low (0.8 ± 0.8%). In terms of frequency of use, the majority of households reported that they collect plants from all land-use types on a daily to weekly basis for all use categories except for construction and weaving (Table S6, Fig. 4). Concerning purpose of use, households collected plants from all land-use types almost exclusively for subsistence (i.e. use only); people rarely sold collected plants (Table S7).

**Fig. 5:**
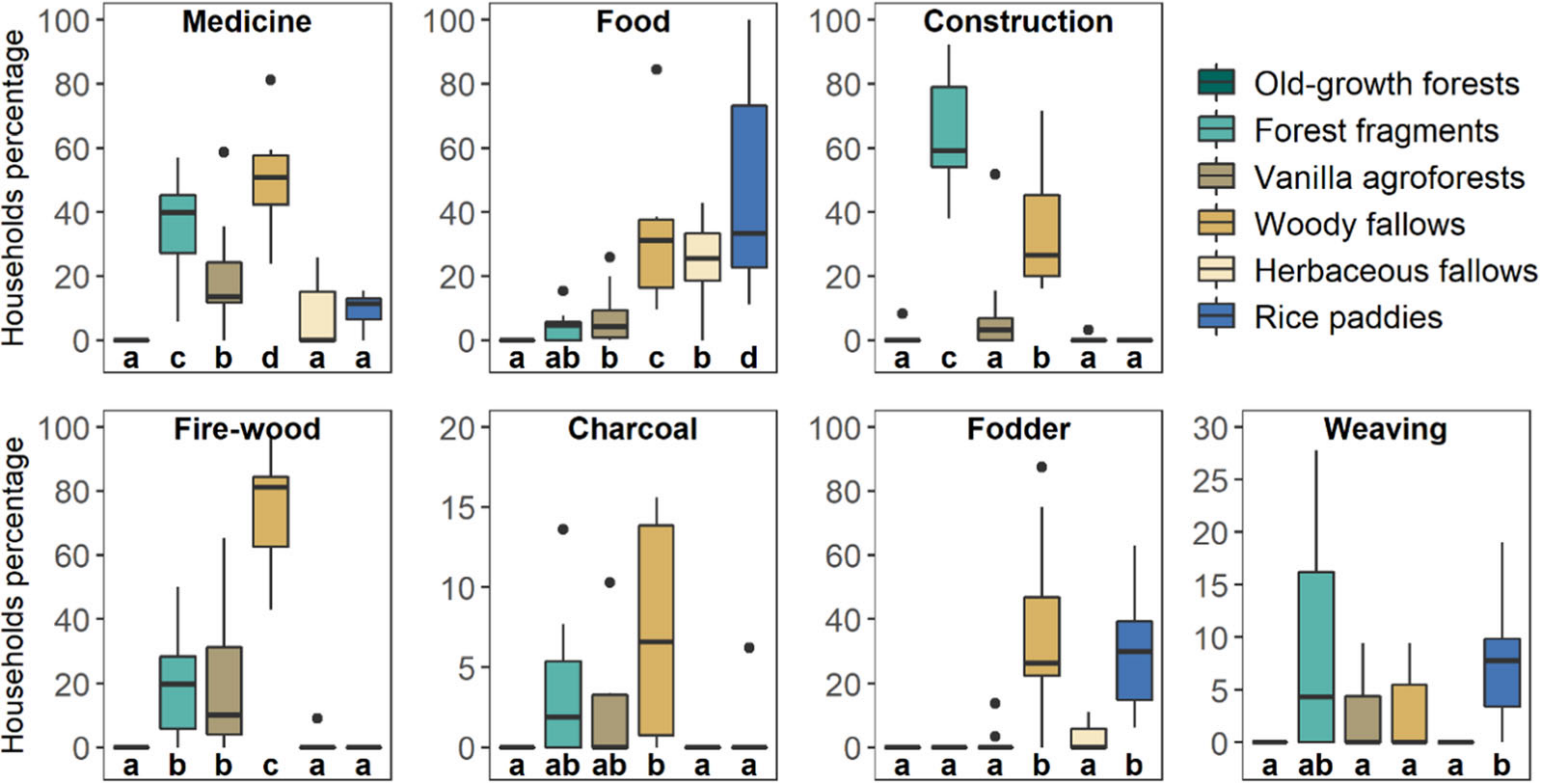
Variation of household percentage collecting plants for seven categories of plant use in north-eastern Madagascar. For the explanation of a boxplot and letters see the caption of Fig. 3. Note the different scaling of the y-axes

When comparing land-use types for each use category, we found that, for collecting plants for medicine, woody fallows (50.7 ± 4.8%) and forest fragments (34.2 ± 5.5%) were the most cited land-use types by households (Table S8; Fig. 5). Rice paddies had the highest percentage of households which reported collecting plants for food (excluding rice; 46.2 ± 10.5%) followed by woody fallows (31.7 ± 6.8%). Forest fragments were the most reported location to collect plants for construction (64.6 ± 5.6%; Fig. 4). Woody fallows were the main location to collect plants for firewood (74.1 ± 5.5%) and charcoal (7.4 ± 2.1%). For fodder, woody fallows and rice paddies were the main location of collection (35.5 ± 8.8% for woody fallows and 31 ± 6.3% for rice paddies; Table S6). Forest fragments (8.6 ± 3.3%) and rice paddies (7.4 ± 1.9%) were the main land-use type to collect plants for weaving materials. Compared to vanilla agroforests, woody fallows were more frequently reported by households as a place to collect plant species for all categories of use, except for weaving (Fig. 5).

### Species used by households across land-use types

In total, households used 364 local names of plants, of which 21 could not be identified. Amongst the remaining 343 local names, we determined 285 species/morpho-species, comprising 85 endemics (30%) and 159 non-endemics (56%). Amongst non-endemic plants, 79 (28% of total species) were native and 80 (28%) exotics. We could not identify the origin of 41 morpho-species (14%). Almost 70% (193) of the overall species used were collected from woody fallows, whilst 46% (132) from forest fragments (Figure S4).

**Fig. 6:**
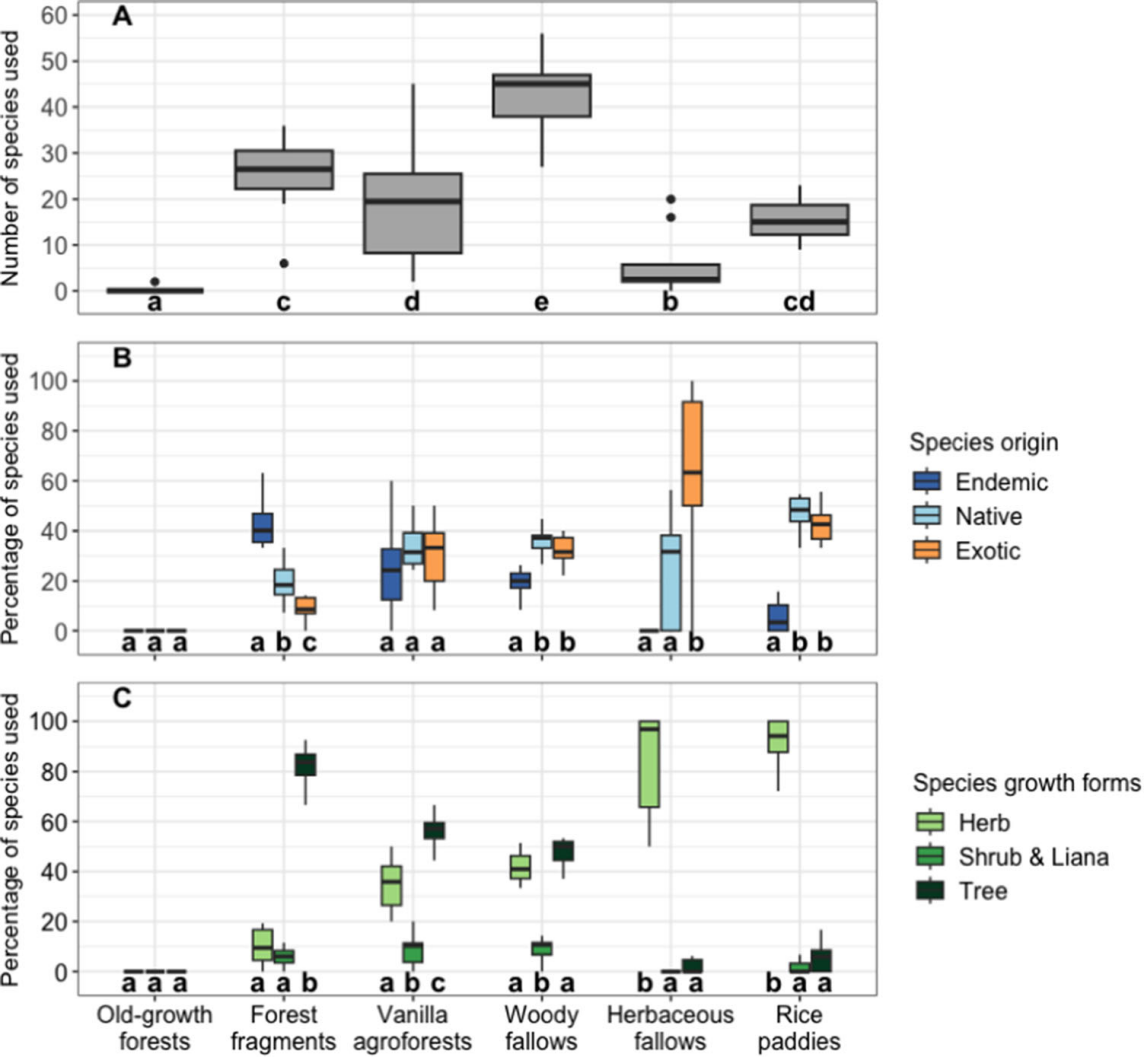
Overview of the diversity of plant species used by rural households across the prevalent land-use types in north-eastern Madagascar based on the number of species used by households per village (**A**), the percentage of species used classified for their origin (**B**), and their growth forms (**C**). Letters represent the results of a post hoc test with *p* < 0.05 for a significant difference between land-use types for (**A**) and between groups (growth form or species origin) within a land-use type for (**B**) and (**C**). For the explanation of boxplot characteristics, see the caption of Fig. 3

Woody fallows were the land-use type that had the highest number of plant species used per village ([mean number of species used per village ± SE] = 49.2 ± 6.2), followed by forest fragments (25.6 ± 2.8), vanilla agroforests (19.3 ± 4.3), and rice paddies (15.5 ± 1.4). The lowest number of species used was in old-growth forests (0.2 ± 0.2, Table S9–S10) and herbaceous fallows (5.7 ± 2.1; Fig. 6A). In terms of species origin, most of the species collected from forest fragments were endemic or native (Fig. 6B; Table S11). Species from vanilla agroforests were a mix of endemic, native, and exotic species, whereas woody fallows were mainly natives and exotic with some endemics (Fig. 6B; Table S11). For herbaceous fallows, the species were predominantly exotic, whilst for rice paddies, species were also mainly non-endemics, i.e. native and exotic (Fig. 6B; Table S11). In terms of growth forms, more than 80% of species collected from forest fragments were trees (Table S11; Fig. 6C). For woody fallows, the species collected were a mix of different growth forms, similarly to vanilla agroforests (Fig. 6C). Herbaceous fallows and rice paddies provided mainly herbs (Table S11; Fig. 6C).

The most commonly used tree species were collected from woody fallows, forest fragments, as well as vanilla agroforests (Figure S5). These species were mostly used by the households on a daily to weekly basis for firewood, charcoal (e.g. *Clausena excavata* and *Croton argyrodaphne)* and medicine (e.g. *Burasaia madagascariensis* and *Tabernaemontana mocquerysii*; Figure S6–S7) and on an annually to more rarely basis for construction (e.g. *Faucherea* sp. and *Cleistanthus* sp., Figure S6–S7). The most reported shrub and liana species were collected by households on a daily to weekly basis from woody fallows which were endemic and mainly used for medicine (e.g. *Acalypha filiformis* and *Triclisia calopicrosia*) and firewood (e.g. *Psorospermum fanera*). For herbs, most cited species were collected primarily from woody fallows, rice paddies, and herbaceous fallows (Fig. S6–S7). The majority of the most reported herb species were mostly non-endemics (exotic or native) and were mainly used on a daily to weekly basis for fodder (e.g. *Stenotaphrum dimidiatum* and *Sorghum arundinaceum*), food (e.g. *Ipomoea aquatica* and *Solanum americanum*), and medicine (e.g. *Mimosa pudica* and *Lygodium lanceolatum)* and on an annually to more rarely basis for weaving (e.g. *Lepironia articulata* and *Eleocharis dulcis*, Fig. S6–S7).

## Discussion

Our study showed that 88% of all rural households benefited from provisioning ecosystem services in the landscape mosaics of north-eastern Madagascar, whilst we also found a great variation in the services and products provided by each land-use type. Old-growth forests and forest fragments were perceived as particularly important for ensuring regulating services mostly by female-headed, better-educated, and younger households, whilst fallow lands, vanilla agroforests, and rice paddies were perceived most important for provisioning services by households of different socio-demographic groups. Forest fragments were primarily used to collect plants materials for construction and weaving on an annually to more rarely basis, whilst woody fallows were used for medicine, firewood, charcoal as well as food collection on a daily to weekly basis. Herbaceous fallows and rice paddies were mainly used for collecting plants on a daily to a weekly basis for food. Woody fallows had the highest number of used species followed by forest fragments. Species collected from forest fragments were mainly endemic, whilst those collected from woody fallows were a mix of different origins. As for growth forms, species from forest fragments were mostly trees, whereas woody fallows provided diverse products ranging from herbs and shrubs to trees. Our results shed new light on the high importance of lands under shifting cultivation to rural households, specifically woody fallows, which were previously regarded as wasteland or of little importance for ecosystem services (Peña Valderrama 2020). Moreover, these results provide evidence that each land-use type has certain complementary specific benefits (e.g. water and climate regulation, providing medicine, food, firewood, or fodder) making the diversity of land uses within a mosaic landscape an important asset for rural livelihoods.

### Fallow lands benefits: multiple services

Fallow lands (i.e. woody fallows and herbaceous fallows) represented the most commonly owned or accessed land-use types (more than 90% of households own woody fallow) and were commonly used as a place to collect plants on a daily to weekly basis for different purposes. Woody fallows were reported to contribute more to provisioning services to households compared to other land-use types, especially in terms of firewood, medicine, as well as fodder and charcoal. This indicates that in addition to rice production and food security (Andriamparany et al. 2021), shifting cultivation lands contribute substantially to the livelihoods of rural households through energy, medicine, food diversification, and animal husbandry, which is in line with the findings of Zaehringer et al., (2017) in Madagascar, Ambrose-Oji (2003) in Cameroon, and Pouliot and Treue (2013) in Ghana and Burkina Faso. Therefore, fallow lands are valuable for the daily life of rural households, despite their lower perceived importance compared to forests for other ecosystem services, such as carbon storage (De Beenhouwer et al. 2013; Peña Valderrama 2020; Soazafy et al. 2021) or water regulation (Sannigrahi et al. 2018). This highlights the importance of looking into a wider array of ecosystem services as well as a specific context to understand the importance of ecosystems (Sayer et al. 2013; Quintas-Soriano et al. 2018). Moreover, fallow lands provided the highest number of species to households (an average of 50 species per village and 193 species in total), most likely because natural forests have decreased significantly in the study region during the last six decades due to shifting cultivation (Curtis et al. 2018; Vieilledent et al. 2018). Fallow lands now dominate the landscape (Zaehringer et al. 2016), thus people rely on fallow lands for many provisioning services due to their accessibility (Pouliot and Treue 2013).

### Ecosystem services benefits and trade-offs across forests and land conversions

Forests were perceived by rural households to be essential for ensuring regulating services, but shifting cultivation lands or agroforests were also perceived as important, as they ensure provisioning services. This indicates that converting forests into shifting cultivation lands or vanilla agroforests is perceived to be reducing the ecosystem’s ability to ensure regulating services, for example, water regulation (Díaz et al. 2006; Steffan-Dewenter et al. 2007). However, households benefit from plant materials and agricultural yields from land uses following forest conversion. This trade-off between regulating and provisioning services happens when natural forests are converted into agricultural lands as the transformation affects ecosystem processes (Millennium Ecosystem Assessment 2005; Clough et al. 2016; Dade et al. 2019), which needs to be balanced.

In terms of species growth forms, species used from forest fragments were mostly trees, whilst vanilla agroforests and woody fallows provided a mix of trees and herbs with some shrubs and lianas. Herbaceous fallows and rice paddies were, on the contrary, used mainly to collect herb species. Hence, converting forests into shifting cultivation lands or agroforestry systems is related to changes in the growth forms of the plants collected by households, from predominantly trees in forests, over a mix of trees and herbs in woody fallows and vanilla agroforests, to strictly herbs in herbaceous fallows. These changes in used plant growth forms follow the shift of plant community compositions and structure across these land-use types (Osen et al. 2021; Raveloaritiana et al. 2021).

Woody fallows outperformed vanilla agroforests in ecosystem service provision, as they provided more species (50 species vs. 20 in vanilla agroforests) and covered all use categories (except weaving). Thus, transforming fallow lands into vanilla agroforests provides a high-income opportunity through vanilla cash cropping (Martin et al. 2022), but at the cost of fundamental services provided by plants for medicine, energy, food, building materials, and livestock. These trade-offs indicate that the ownership of multiple land-use types serves as a safety net that ensures access to regulating, provisioning, and cultural ecosystem services as well as agricultural output for rural households (Swinton et al. 2007; Vialatte et al. 2019).

### Plant uses and conservation

In total, we found 285 different species used by households across all villages with 30% endemic, 56% non-endemic (28% native and 28% exotic) and 14% unknown origin. The species used from fallows lands, vanilla agroforest, and rice paddies were mainly non-endemics, i.e. natives and exotics. Additionally, non-endemic species were often reported and used for all categories (medicine, food, construction, firewood, charcoal, fodder, and weaving). These results indicate that non-endemic species contribute more to rural livelihoods than endemics, most likely because most of the plant species outside of protected areas are non-endemics (Raveloaritiana et al. 2021). Endemic species conservation is of high priority in Madagascar, but here, the use of native and exotic species for diverse use categories highlights the importance of non-endemic plants, especially exotics, to support rural livelihoods (Kull et al. 2014). This viewpoint needs to be better integrated into land-use planning, thereby acknowledging the needs of people (Simberloff et al. 2013; Courchamp et al. 2017).

Most of the species used from forest fragments are endemic. Households used some endemic species from woody fallows as well. Thus, conservation of endemic plants in the agricultural matrix is not only important for conservation but benefits people; hence, traditional species-based conservation can be integrated into the ecosystem services framework. This integration can provide additional arguments for the conservation of plant species (Mace et al. 2012), as the loss of species has direct consequences for ecosystem functions (Fox and Harpole 2008) and human well-being (Díaz et al. 2006).

### Study limitations

We used households’ percentages and perceptions as a measure to evaluate the importance of each land-use type for ecosystem services. We acknowledge that our measure does not quantify the actual services provided by these land-use types to households. For instance, households often mentioned woody fallows as a place to collect plants for different purposes, but the quantity of the products per area might be lower compared to forests. Furthermore, the abundance of species may be more important for ecosystem services than species richness (Winfree et al. 2015). Quantification of the plants used by households in their respective lands would have provided more specific information, important for identifying species overexploitation.

Provisioning and regulating services were more often reported by the households than the supporting and cultural services. This is probably because we used open-ended questions about the perception of ecosystems services and the interviewee may have focused more on direct or more obvious benefits (provisioning and regulating services such as materials for food, water regulation) than the indirect ones (cultural services like spaces for ceremonies or recreation; Lhoest et al. 2019). More targeted questions, such as asking about the types of ecosystem services one by one, or the use of an approach to scale responses, or the use of a participatory method, would have helped the interviewees to provide more indirect benefits and reveal the values of nature more in depth (Lhoest et al. 2019), but at the cost of potential biases in their individual perception caused by targeted questions, confusion over ranking options or group discussions (Neuman 2014).

As part of shifting cultivation practices, farmers leave their lands fallow for a certain period to regenerate the soil quality and control unwanted weeds (Nye and Greenland 1960; Zaehringer et al. 2018). However, amongst regulating services, soil quality was rarely reported as a benefit of fallow lands. This could be because these fallow lands may lay fallow in waiting for agricultural activities other than shifting cultivation (Martin et al. 2023), thus benefiting from or increasing soil quality may not the primary reason for fallowing. In addition, the household did not report the soil quality associated with fallows as they may have focused more on provisioning ecosystem services during the interviews.

Despite a relatively high percentage of households that mentioned old-growth forests as a location to collect plants for construction, only two species’ names were reported as collected from this land-use type. This could be because respondents might have been hesitant to admit the use of species from old-growth forests which are not permitted due to their status as protected areas (IUCN 1994). Therefore, the respondent might have mentioned other land-use types as locations to collect these types of species or did not mention the species at all to avoid self-incrimination; a pattern observed in a study on lemurs hunting elsewhere in Madagascar (Borgerson et al. 2018). This may limit the validity of our results concerning the use of plants from old-growth forests. Here, a specialised method for investigating sensitive behaviours (e.g. randomised response technique) could be used to generate more accurate, but potentially less specific results (Razafimanahaka et al. 2012). Another possible reason why households reported deriving few provisioning ecosystem services from old-growth forests is that most study villages were far away from the remaining old-growth forests (see Fig. S1), hence households relied more on the land-use types in their surroundings to collect plants for their daily lives.

### Rural livelihoods and landscape management implications

Ensuring sustainable agriculture and use of natural resources to alleviate poverty, achieve zero hunger, and improve quality of life are the most prominent and challenging goals for rural communities in Madagascar (Zaehringer et al. 2018). Within mosaic landscapes in north-eastern Madagascar, shifting cultivation and irrigated rice field are important for producing food for subsistence, hence food security, whilst agroforestry systems are essential in producing cash crops (Zaehringer et al. 2017; Llopis et al. 2021; Martin et al. 2022). Our results revealed that, beyond crop production, rural households depend on diverse sets of services from their surroundings to support their livelihoods. This confirms that the natural environment is the centre of rural livelihoods in Madagascar (Angelsen et al. 2014; Scales 2014a), calling for strategies that balance biodiversity conservation goals with local people needs (Pascual et al. 2021). Thus, understanding the importance of mosaic landscapes and their ecosystem services for rural households is a basis for effective land-use planning for both improved livelihoods and conservation.

Our findings show that forests are needed to complement the role of shifting cultivation lands in ecosystem services provision within the agricultural landscapes. Maintaining remaining forest fragments and old-growth forests is still most important to mitigate trade-offs between the conservation of biodiversity and regulating services on the one hand and provisioning services from different land-use types on the other hand (Llopis et al. 2021; Martin et al. 2022). Furthermore, female-headed, better-educated, and younger households perceived regulating services of old-growth forests to be more important than households headed by males or younger or less-educated persons. Hence, promotion of females or young people in land-use planning, as well as improved education for males, younger, and less-educated persons, appears to be essential to promote sustainable trade-offs between conservation and land use in landscape management. This indicates that households’ perception of the importance of the old-growth forests and forest fragments in terms of ecosystem services, namely regulating and supporting services, depends on their socio-demographic groups (Quintas-Soriano et al. 2018). Moreover, these patterns could also be associated with people’s exposure to information campaigns about their natural environment. Thus, more environmental education with consideration of socio-demographic groups could benefit forest conservation by increasing the importance of these land-use types and mitigating the existing trade-offs.

During the last six decades, the forest cover in north-eastern Madagascar has decreased significantly (Vieilledent et al. 2018) and landscapes are now dominated by shifting cultivation (Zaehringer et al. 2016; Curtis et al. 2018). Consequently, the importance of fallow lands for the local communities might have increased, as these ecosystems provide plants for different purposes, namely medicine, food, energy, and construction for the rural communities. However, shifting cultivation is also the main driver of deforestation in Madagascar (Scales 2014b) and may cause land degradation if under unsustainable management (Styger et al. 2007). Thus, an option would be to promote farming diversification that can maintain essential ecosystem services, yields, and biodiversity (Tamburini et al. 2020). As an alternative option, a longer fallow period combined with active tree plantings for use or restoration (depending on households’ needs) can provide further services to households, whilst the soil recovers from nutrient depletion. Nonetheless, putting land aside depends also on having sufficient land for the subsistence of the household, which itself depends on household size. The best management of fallows would be then to actively restore degraded lands formerly under shifting cultivation by promoting a diversity of plant species to reduce pressures on forest fragments and to avoid overexploitation of protected plant species, ensuring continuous access to provisioning ecosystem services. Including private lands, especially fallow and degraded lands, into restoration programmes may enable a bottom-up restoration approach, which scales from local goals (improving livelihoods) to a global agenda (planting trees in the scope of the Bonn Challenge to increase carbon sequestration) and improves outcomes for people and biodiversity alike (Holl 2017). Such strategies will be fundamental in ensuring sustainable land-use planning that empowers local communities by taking into account their needs and aspirations. This will contribute to larger-scale goals of sustainable rural development (de Groot et al. 2010; Costanza et al. 2014).

Our results confirmed that mosaic landscapes consisting of multiple land-use types are required to provide ecosystem services for rural livelihoods and maintain plant biodiversity. Here, the diverse land-use types that form the mosaic landscapes of north-eastern Madagascar are simultaneously used by households, complementing each other in ensuring services for rural livelihoods. Despite this, narratives, social norms, and cultural legacies can continue to influence the land-use decisions of young Malagasy people living in rural areas, where slash-and-burn cultivation may be used to acquire lands they need as they are unable to inherit lands, whilst their parents are still alive (Fulgence et al. 2020; Jones et al. 2022). Moreover, land tenure legislation and processes in Madagascar are complex and often being the first to use and reclaim a land may lead to customary ownership (André Teyssier 2010). This could further explain the ownership of multiple types of lands which then maintains the mosaic of land uses in Madagascar. Therefore, land-use policies should consider the local contexts around the land tenure and the complementarity of ecosystem services from these land-use types to ensure the sustainability of mosaic landscapes, providing diverse sets of benefits for both people’s daily life and biodiversity conservation (Kremen and Merenlender 2018; Pascual et al. 2021).

## Conclusion

The results of our study show that each of the prevalent land-use types provides a unique set of ecosystem services for rural households. Old-growth forests were perceived as being important for regulating and supporting services, whilst rural households valued the provisioning of ecosystem services from other land-use types. Forest fragments stood out as a land-use type that provided timber for construction and with a high percentage of trees as well as endemic species within mosaic landscapes. Woody fallows, the most widely accessible land-use type, were perceived as highly important to collect plant species mostly daily, offering the highest richness of useful plant species of all land-use types for manifold purposes, especially for medicine, energy, and food. Fallow lands, forest fragments, and old-growth forests were therefore complementary in providing ecosystem services to local communities. This highlights how agricultural landscapes with multiple land-use types in combination with protected areas are important to preserve biodiversity and sustain livelihoods. In addition, fallow lands, a land-use type so far seen as unimportant for ecosystem services, provided multiple and important ecosystem services, contributed significantly to rural livelihoods, and were intensively used by rural households, despite supporting much less biodiversity than forests. Thus, our findings advocate for considering fallow lands in land management and conservation strategies. Including fallow lands in land-use planning in tropical mosaic landscapes will then ensure the wealth of ecosystem services from diverse land uses, not only for conservation purposes but also to support rural livelihoods.

## Supplementary Information

Below is the link to the electronic supplementary material.

Supplementary file1 (PDF 2648 KB)

## Data Availability

The complete survey questionnaire and the data used in this study are stored in the Open Science Framework data repository (https://osf.io/7ca5g/).
