# Supplementary material for: Complementary ecosystem services from multiple land uses highlight the importance of tropical mosaic landscapes
Source: Ambio. 2023 Jun 7;52(10):1558–74. doi: 10.1007/s13280-023-01888-3 (PMC10460756; doi:10.1007/s13280-023-01888-3)
Supplement: Supplementary file 1 — Supplementary file1 (PDF 2648 KB) [file 13280_2023_1888_MOESM1_ESM.pdf]

Ambio
Supplementary Information
This supplementary information has not been peer reviewed.

Title: **Complementary ecosystem services from multiple land uses highlight the importance of tropical mosaic landscapes**

Authors: Estelle Raveloaritiana[1,2,3], Annemarie Wurz[2,4], Kristina Osen[5], Marie Rolande Soazafy[5,6,7], Ingo Grass[8], Dominic Andreas Martin[9, 10], Claudine Bemamy[11], Hery Lisy Tiana Ranarijaona[7], Cortni Borgerson[12], Holger Kreft[9,13], Dirk Hölscher[5,13], Bakolimalala Rakouth[1], Teja Tscharntke[2,13]

Affiliations:
[1] Plant Biology and Ecology Department, University of Antananarivo, Antananarivo, Madagascar
[2] Agroecology, Department of Crop Sciences, University of Goettingen, Göttingen, Germany
[3] Sustainable Agricultural Systems and Engineering Laboratory, Westlake University, Hangzhou, China
[4] Conservation Ecology, Department of Biology, Philipps-Universität Marburg, Marburg, Germany
[5] Tropical Silviculture and Forest Ecology, University of Goettingen, Göttingen, Germany
[6] Natural and Environmental Sciences, Regional University Centre of the SAVA Region (CURSA), Antalaha
[7] Natural Ecosystems (EDEN), University of Mahajanga, Mahajanga, Madagascar
[8] Ecology of Tropical Agricultural Systems, University of Hohenheim, Stuttgart, Germany
[9] Biodiversity, Macroecology and Biogeography, University of Goettingen, Göttingen, Germany
[10] Department of Geography, University of Zurich, Zurich, Switzerland
[11] Diversity Turn in Land Use Sciences Research Project, Sambava, Madagascar
[12] Department of Anthropology, Montclair State University, Montclair, USA
[13] Centre for Biodiversity and Sustainable Land Use (CBL), University of Goettingen, Göttingen, Germany

Name and present address of the corresponding author:
Estelle Raveloaritiana, Sustainable Agricultural Systems and Engineering Laboratory, School of Engineering, Westlake University, Hangzhou, China
Email: eraveloaritiana@gmail.com

I. Details about methods

1. Details about village selection

To select these 10 out of the 60 villages from Hänke et al., (2018), we first did a cartographic stratification by eliminating villages where the landscapes within a 2 km radius were dominated by wetlands or lakes, rice fields, and coconut plantations, which were too close to the ocean or harboured no forest fragments. From the resulting 17 villages, we considered the distance between them and randomly removed one of the villages for village pairs with <4 km distance. After this second step, we had 14 villages that we visited; one of them did not have all the prevalent land-use types and the community of another village did not want to participate in our study. With the 12 remaining villages, we then randomly selected the 10 villages as a compromise between the number of villages and logistical constraints.

Reference:

Hothorn, T., F. Bretz, and P. Westfall. 2008. Simultaneous inference in general parametric models. *Biometrical Journal* 50: 346–363. doi:10.1002/bimj.200810425.

Wickham, H. 2016. *ggplot2: Elegant Graphics for Data Analysis*. Springer-Verlag New York.

2. Categorisation of benefits into ecosystem services categories (Millennium Ecosystem Assessment, 2005)

Regulating services: air quality; environmental quality; soil humidity; soil quality; erosion protection; water regulation

Supporting services: wild animals' habitat

Provisioning services: clean water; food; fruit trees; honey; medicinal plants; firewood; charcoal wood; liana for string; plants for construction; weaving materials; cattle fodder;

Cultural services: land for descendants; recreation

- Millennium Ecosystem Assessment. 2005. Ecosystems and human well-being: biodiversity synthesis. World Resources Institute, Washington, DC.

3. Details about data analysis:

To determine the value of land-use types for ecosystem services, we calculated the mean of household percentages that reported each ecosystem service type within each land-use type with standard errors. Then, we visualised the mean percentage of the households benefiting from each type of ecosystem services per land-use type, using polar area charts

with R-package *ggplot2* (Wickham 2016). Additionally, we also characterised the households relying on different land uses for each type of ecosystem services by categorising households' heads by gender (male and female), age group (young adults <36 years old; middle-aged adults: 36-55 years old and older adults > 55 years old) and highest attained educational level (no school education, primary school, secondary school, and high school or university). For this, we analysed the variation of the groups for each type of ecosystem service within each land-use type, using generalised linear models with a binomial distribution.

To depict the differences between land-use types for each ecosystem service type, we analysed the variation of the percentages of households reporting enjoying each type of ecosystem service across land-use types using generalised linear models with binomial distribution followed by post-hoc tests using the R-package *multcomp* (Hothorn et al. 2008).

To evaluate the importance of each land-use type for each use category, we calculated the percentage of households that reported collecting plants from the respective land-use type along with their frequency of use as well as their purposes (use, selling, or both). Subsequently, we used the Sankey diagram from the *ggforce* R-package for visualisation. We also analysed the variation in the percentage of households that collected plants across land-use types by applying generalised linear models with binomial distribution followed by post-hoc tests.

To assess the number of species used, we first calculated the total number of species reported by all interviewed households, and the total number of species from each land-use type. To detect the importance of each land-use type in terms of the number of species used at the village level, we calculated: (1) the number of species used; (2) the percentage of endemic, native, or exotic species used; and (3) the percentage of growth forms (tree, shrub, liana and herb) used. To evaluate the variation in the species used across land-use types, we also applied a generalised linear model with generalised Poisson distribution for the number of species used and binomial distribution for species origin and growth form groups. We also did post-hoc tests to determine the differences between land-use types and between species origin and growth form groups within each land-use type.

To determine where most cited species were collected across the land-use types, we calculated the number of households naming each species (hereafter called citation number)

per land-use type. For this, we used the fractional citation number, i.e. if a species was cited once for one use category by one household but collected from three different land-use types, the citation for each land-use type is one-third. However, when the species is mentioned once and collected from a single land-use type then the citation is one. Then, we visualised the species by summing up their fractional citation number per land-use type for all households using ranked bar plots separated by the growth forms (tree, shrub, liana and herb).

References:

Hothorn, T., F. Bretz, and P. Westfall. 2008. Simultaneous inference in general parametric models. *Biometrical Journal* 50: 346–363. doi:10.1002/bimj.200810425.

Wickham, H. 2016. *ggplot2: Elegant Graphics for Data Analysis*. Springer-Verlag New York.

4. Reference for icons in Figure 2

Adrien Coquet (2020) Medicine icon retrieved from https://thenounproject.com/search/?q=Medicine&i=1658521

Vectorstall (2020) Food icon retrieved from https://thenounproject.com/search/?q=Vegetable&i=2764240

Prau Sindoro (2020) Construction icon retrieved from https://thenounproject.com/search/?q=construction+wood&i=3547117

arif fajar yulianto (2020) Firewood icon retrieved from https://thenounproject.com/search/?q=Fire+wood&i=1400010

Eucalyp (2020) Charcoal icon retrieved from https://thenounproject.com/search/?q=Charcoal&i=3051225

H V P (2020) Fodder icon retrieved from https://thenounproject.com/search/?q=Fodder&i=831760

Amrita Mayuri (2020) Weaving icon retrieved from https://thenounproject.com/search/?q=Weaving&i=858481

Table S1. Overview of the questionnaire used to evaluate the importance of land-use types in terms of ecosystem services types (I) and the collection of plants for the 7 categories of use (II). Full questionnaire of the survey is available at Open Science Framework data repository (https://osf.io/7ca5g/). *Prior to each interview, CIB started with a self-introduction, along with the objective and structure of the survey, the approximate length of the interview, and the use of the data (academic and research purposes). The interviewer also ensured the respondent the anonymity of their identity in any use of the data and then asked verbally for their consent to participate in the survey.*

**I) Importance of land-use types for farmers (for each land-use type):** old-growth forests, forest fragments, vanilla agroforests, woody fallow, herbaceous fallows, and rice paddies.	**II) Importance of land-use type to collect plants for medicine/ food/ construction/ firewood/ charcoal/ fodder/ weaving**
1. Do you own or have access to old-growth forests, forest fragments, vanilla agroforests, woody fallows, herbaceous fallows and rice paddies?<br>● No access<br>● I have access but not on my own (it can belong to their relatives or neighbour who granted access)<br>● I possess this land-use type<br><br>2. What do you use for what or what benefits do you get from the land-use type (other than the main crop for rice paddies and vanilla agroforests)?	1. Please name plant species you use for each of the following categories: medicine, food, construction, firewood, charcoal, fodder and weaving<br>For each named species:<br>2. Is it for use only, selling only or for both use and selling?<br>3. Where do you collect this plant and how often?<br>● Old-growth forests<br>● Forest fragments<br>● Vanilla agroforests<br>● Woody fallows<br>● Herbaceous fallows<br>● Paddy rice<br>● Other (e.g. border of the river, lake, around the village, market)

Table S2. Total number of households that have access and/or own land-use types across all villages

Land-use types	Overall Access %HH	Access only %HH	Own %HH
Old-growth forests	29.2 ±3.2	29.2 ±3.2	-
Forest fragments	56 ±6.6	31.6 ±4.7	24.4 ±3.8
Vanilla agroforests	88.4 ±4.8	3.1 ±0	87.8 ±4.9
Woody fallows	95 ±3.4	19.8 ±5.4	77.2 ±6.3
Herbaceous fallows	51.8 ±9.3	3.8 ±0.5	48.4 ±9.3
Rice paddies	92.2 ±3.4	6.6 ±1.7	86.9 ±3.8

Table S3. Predicted percentage of households mentioning benefits from land-use types in each category of ecosystem services and the overall percentage of the category with standard errors from model prediction results.

ES type	Services/benefits	Old-growth forests	Forest fragments	Vanilla agroforests	Woody fallows	Herbaceous fallows	Rice paddies
Individual regulating services	Air quality	13.2 ±3.1	2.3 ±1	-	-	-	-
	Environmental quality	5.8 ±3.4	0.4 ±0.4	-	-	-	-
	Soil humidity	19.4 ±10.6	6.8 ±4	-	-	-	-
	Soil quality	2.6 ±1.8	2.2 ±1.3	-	0.3 ±0.3	-	-
	Erosion protection	0.4 ±0.4	-	-	-	-	-
	Water regulation	53.1 ±9.7	13.6 ±5.6	-	-	-	-
**Overall regulating services**		**77 ±3.6**	**22.1 ±5.2**	**-**	**0.3 ±0.3**	**-**	**-**
Individual supporting services	Animals' habitat	9.4 ±1.8	3.5 ±1.5	-	-	-	-
**Overall supporting services**		**9.4 ±1.8**	**3.5 ±1.5**	**-**	**-**	**-**	**-**
Individual provisioning services	Clean water	23.2 ±9.9	6.2 ±3.3	-	-	-	-
	Unmanaged cultivated food	-	-	35.8 ±4.5	51.8 ±7.7	58.6 ±5.2	4.3 ±1.3
	Wild Food	0.9 ±0.6	0.4 ±0.4	2.5 ±1.1	0.6 ±0.6	1 ±1	0.3 ±0.3
	Fruit trees	0.8 ±0.8	0.4 ±0.4	43.7 ±4.3	4.8 ±1.8	0.7 ±0.7	-
	Honey	0.4 ±0.4	-	-	-	-	-
	Medicinal plants	2.2 ±1.2	0.7 ±0.7	1.8 ±0.8	0.3 ±0.3	-	-
	Firewood	3.2 ±1.6	3.8 ±2.3	2.8 ±0.8	6 ±2.7	-	-
	Charcoal wood	-	0.4 ±0.4	0.3 ±0.3	-	-	-
	Liana for string	-	1.8 ±0.7	-	-	-	-
	Plants for construction	28.8 ±4.4	24.4 ±2.9	1.6 ±0.7	1.8 ±0.8	-	-
	Weaving materials	-	1.1 ±1.1	-	-	-	-
	Zebu fodder	-	-	-	-	6.8 ±3	0.3 ±0.3
**Overall provisioning services**		**53.4 ±7.5**	**32.2 ±5.2**	**62.6 ±4.6**	**60.6 ±6.1**	**62.8 ±5.6**	**5 ±1.4**
Individual cultural services	Land for descendants	-	9.2 ±2.8	-	1.3 ±0.7	-	-
	Recreation	0.4 ±0.4	-	-	-	-	-
**Overall cultural services**		**0.4 ±0.4**	**9.2 ±2.8**	**-**	**1.3 ±0.7**	**-**	**-**

- Animals' habitat: shelter for any type of animal from an ecological point of view
- Unmanaged cultivated food: plants cultivated but do not require any management as they grow with wild plants
- Wild food: wild and edible plants
- Plants for construction: plants used for construction via their woods or leaves, bark etc…
- Land for descendants: land that is reserved to be used by their children or grandchildren in the future

Table S4. Multiple comparisons of land-use types based on their household percentage that benefits for each ecosystem services type at village level in north-eastern Madagascar

	Regulating services						Supporting services				
contrast	estimate	SE	df	t.ratio	p.value		estimate	SE	df	t.ratio	p.value
Old-growth forests - Forest fragments	3.0575	0.503	53	6.082	<.0001		1.431	0.456	53	3.141	0.0311
Old-growth forests - Vanilla agroforests	6.2286	0.626	53	9.958	<.0001		2.096	0.459	53	4.565	0.0004
Old-growth forests - Woody fallows	6.1325	0.625	53	9.817	<.0001		2.096	0.459	53	4.565	0.0004
Old-growth forests - Herbaceous fallows	6.2286	0.626	53	9.958	<.0001		2.096	0.459	53	4.565	0.0004
Old-growth forests - Rice paddies	6.2286	0.626	53	9.958	<.0001		2.096	0.459	53	4.565	0.0004
Forest fragments - Vanilla agroforests	3.171	0.473	53	6.698	<.0001		0.665	0.451	53	1.475	0.6813
Forest fragments - Woody fallows	3.0749	0.473	53	6.5	<.0001		0.665	0.451	53	1.475	0.6813
Forest fragments - Herbaceous fallows	3.171	0.473	53	6.698	<.0001		0.665	0.451	53	1.475	0.6813
Forest fragments - Rice paddies	3.171	0.473	53	6.698	<.0001		0.665	0.451	53	1.475	0.6813
Vanilla agroforests - Woody fallows	-0.0961	0.452	53	-0.212	0.9999		0	0.45	53	0	1
Vanilla agroforests - Herbaceous fallows	0	0.452	53	0	1		0	0.45	53	0	1
Vanilla agroforests - Rice paddies	0	0.452	53	0	1		0	0.45	53	0	1
Woody fallows - Herbaceous fallows	0.0961	0.452	53	0.212	0.9999		0	0.45	53	0	1
Woody fallows - Rice paddies	0.0961	0.452	53	0.212	0.9999		0	0.45	53	0	1
Herbaceous fallows - Rice paddies	0	0.452	53	0	1		0	0.45	53	0	1
	Provisioning services						Cultural services				
	estimate	SE	df	t.ratio	p.value		estimate	SE	df	t.ratio	p.value
Old-growth forests - Forest fragments	1.9351	0.454	53	4.265	0.0011		-1.0781	0.456	53	-2.366	0.01797
Old-growth forests - Vanilla agroforests	0.9074	0.442	53	2.054	0.3267		0.0987	0.452	53	0.218	0.82705
Old-growth forests - Woody fallows	0.9487	0.442	53	2.148	0.2794		-0.23	0.452	53	-0.508	0.61113
Old-growth forests - Herbaceous fallows	0.8783	0.442	53	1.988	0.3625		0.0987	0.452	53	0.218	0.82705
Old-growth forests - Rice paddies	4.3565	0.508	53	8.569	<.0001		0.0987	0.452	53	0.218	0.82705
Forest fragments - Vanilla agroforests	-1.0277	0.434	53	-2.37	0.1857		1.1768	0.456	53	2.582	0.00981
Forest fragments - Woody fallows	-0.9863	0.433	53	-2.277	0.2216		0.8481	0.455	53	1.862	0.06261
Forest fragments - Herbaceous fallows	-1.0568	0.434	53	-2.434	0.1633		1.1768	0.456	53	2.582	0.00981
Forest fragments - Rice paddies	2.4214	0.461	53	5.247	<.0001		1.1768	0.456	53	2.582	0.00981
Vanilla agroforests - Woody fallows	0.0413	0.428	53	0.097	1		-0.3287	0.452	53	-0.727	0.46731
Vanilla agroforests - Herbaceous fallows	-0.0291	0.428	53	-0.068	1		0	0.452	53	0	1
Vanilla agroforests - Rice paddies	3.4491	0.478	53	7.222	<.0001		0	0.452	53	0	1
Woody fallows - Herbaceous fallows	-0.0704	0.428	53	-0.164	1		0.3287	0.452	53	0.727	0.46731
Woody fallows - Rice paddies	3.4078	0.476	53	7.152	<.0001		0.3287	0.452	53	0.727	0.46731
Herbaceous fallows - Rice paddies	3.4782	0.478	53	7.271	<.0001		0	0.452	53	0	1

Table S5. Mean percentage with standard error of households that are collecting plants of different categories from each land-use type in north-eastern Madagascar

Land use types	Old-growth forests	Forest fragments	Vanilla agroforests	Woody fallows	Herbaceous fallows	Rice paddies
Medicine	0 ±0	34.2 ±5.5	20.2 ±5.2	50.7 ±4.8	7.4 ±3.5	9.5 ±1.6
Food	0 ±0	4.3 ±1.5	7.4 ±2.8	31.7 ±6.8	23.4 ±4.6	46.2 ±10.5
Construction	0.8 ±0.8	64.6 ±5.6	8.7 ±5	34.7 ±6.4	0.3 ±0.3	0 ±0
Fire-wood	0 ±0	20 ±5.3	19.2 ±6.7	74.1 ±5.5	0.9 ±0.9	0 ±0
Charcoal	0 ±0	3.5 ±1.4	2 ±1	7.4 ±2.1	0 ±0	0.6 ±0.6
Fodder	0 ±0	0 ±0	1.7 ±1.4	35.5 ±8.8	3.2 ±1.5	31 ±6.3
Weaving	0 ±0	8.6 ±3.3	2.3 ±1.1	2.5 ±1.1	0 ±0	7.4 ±1.9

Table S6. Mean percentage with standard error of households that are collecting plants for different categories of use with their frequency of use for households across land use types in north-eastern Madagascar

Land-use types	Frequency of use	Old-growth forests	Forests fragments	Vanilla agroforests	Woody fallows	Herbaceous fallows	Rice paddies
Medicine	Annually to more rarely	-	0.9 ±0.6	-	0.7 ±0.4	-	-
	Monthly to semi-annually	-	0.5 ±0.5	0.6 ±0.4	-	-	-
	Daily to weekly	-	32.8 ±5.3	19.6 ±5.1	50 ±4.6	7.4 ±3.5	9.5 ±1.6
Food	Annually to more rarely	-	0.4 ±0.4	-	4.2 ±1.7	1.1 ±1.1	6.6 ±2.2
	Monthly to semi-annually	-	0.9 ±0.6	1.6 ±0.7	12 ±3.9	3.2 ±1.3	16.4 ±4.4
	Daily to weekly	-	3.1 ±1.6	5.7 ±2.3	15.4 ±3.8	19.1 ±4.1	23.2 ±6
Construction	Annually to more rarely	0.8 ±0.8	63.7 ±5.5	8.7 ±5	33.1 ±6.6	0.3 ±0.3	-
	Monthly to semi-annually	-	-	-	1.3 ±0.5	-	-
	Daily to weekly	-	0.8 ±0.6	-	0.3 ±0.3	-	-
Fire-wood	Annually to more rarely	-	-	-	-	-	-
	Monthly to semi-annually	-	-	-	-	-	-
	Daily to weekly	-	20 ±5.3	19.2 ±6.7	74.1 ±5.5	0.9 ±0.9	-
Charcoal	Annually to more rarely	-	0.9 ±0.9	1 ±0.7	1.9 ±0.8	-	-
	Monthly to semi-annually	-	-	-	-	-	-
	Daily to weekly	-	2.6 ±0.9	1 ±0.5	5.6 ±1.9	-	0.6 ±0.6
Forage	Annually to more rarely	-	-	-	0.3 ±0.3	-	0.3 ±0.3
	Monthly to semi-annually	-	-	-	-	-	-
	Daily to weekly	-	-	1.7 ±1.4	35.2 ±8.6	3.2 ±1.5	30.7 ±6.4
Weaving	Annually to more rarely	-	8.6 ±3.3	2.3 ±1.1	2.2 ±1.1	-	6.4 ±1.9
	Monthly to semi-annually	-	-	-	-	-	0.6 ±0.4
	Daily to weekly	-	-	-	0.3 ±0.3	-	0.3 ±0.3

Table S7. Mean percentage with standard error households that are collecting plants for different categories of use with their purposes for households across land-use types in north-eastern Madagascar

Land-use types	Frequency of use	Old-growth forests	Forests fragments	Vanilla agroforests	Woody fallows	Herbaceous fallows	Rice paddies
Medicine	**Use**	0 ±0	31.9 ±5.5	19.9 ±5.1	50 ±4.6	7.4 ±3.5	9.5 ±1.6
	Use & selling	0 ±0	1.9 ±1	0.4 ±0.4	0.3 ±0.3	0 ±0	0 ±0
	Selling	0 ±0	1 ±1	0 ±0	0.6 ±0.6	0 ±0	0 ±0
Food	**Use**	0 ±0	3.5 ±1	4.9 ±2.5	28.2 ±5.1	21.6 ±4.2	44.9 ±10.7
	Use & selling	0 ±0	0.9 ±0.9	2.7 ±1.5	3.9 ±2.1	2 ±1.1	1.4 ±1.1
	Selling	0 ±0	0 ±0	0 ±0	0 ±0	0 ±0	0 ±0
Construction	**Use**	0.8 ±0.8	54.9 ±5.3	8.7 ±5	33.5 ±6.6	0.3 ±0.3	0 ±0
	Use & selling	0 ±0	10.8 ±2.4	0 ±0	1.4 ±0.6	0 ±0	0 ±0
	Selling	0 ±0	0 ±0	0 ±0	0 ±0	0 ±0	0 ±0
Fire-wood	**Use**	0 ±0	17.9 ±4.9	19.2 ±6.7	73.2 ±5.3	0.9 ±0.9	0 ±0
	Use & selling	0 ±0	2.3 ±1.7	0 ±0	1 ±0.7	0 ±0	0 ±0
	Selling	0 ±0	0 ±0	0 ±0	0 ±0	0 ±0	0 ±0
Charcoal	**Use**	0 ±0	0.5 ±0.5	0.7 ±0.4	1.8 ±0.6	0 ±0	0.3 ±0.3
	Use & selling	0 ±0	0.9 ±0.9	0.4 ±0.4	2.1 ±1	0 ±0	0 ±0
	Selling	0 ±0	3.8 ±2.2	1.7 ±1.2	6.3 ±2.6	0 ±0	0.5 ±0.5
Forage	**Use**	0 ±0	0 ±0	1.7 ±1.4	35.5 ±8.8	3.2 ±1.5	31 ±6.3
	Use & selling	0 ±0	0 ±0	0 ±0	0 ±0	0 ±0	0 ±0
	Selling	0 ±0	0 ±0	0 ±0	0 ±0	0 ±0	0 ±0
Weaving	**Use**	0 ±0	7.6 ±2.9	2.3 ±1	1.9 ±1	0 ±0	6.3 ±1.7
	Use & selling	0 ±0	1.1 ±0.8	0 ±0	0.7 ±0.5	0 ±0	1.2 ±0.6
	Selling	0 ±0	0 ±0	0 ±0	0 ±0	0 ±0	0 ±0

Table S8. Multicomparisons of land-use types based on the household percentage that collects plants for each category of use in North-eastern Madagascar

	Medicine						Food				
contrast	estimate	SE	df	t.ratio	p.value		estimate	SE	df	t.ratio	p.value
Old-growth forests - Forest fragments	-3.3300	0.4620	52	-7.2110	<.0001		-0.731	0.483	52	-1.514	0.657
Old-growth forests - Vanilla agroforests	-2.1800	0.4630	52	-4.7080	0.0003		-0.953	0.486	52	-1.962	0.3777
Old-growth forests - Woody fallows	-4.0300	0.4700	52	-8.5700	< 2e-16		-2.629	0.517	52	-5.085	0.0001
Old-growth forests - Herbaceous fallows	-0.5220	0.4660	52	-1.1200	0.1810		-1.524	0.494	52	-3.087	0.0362
Old-growth forests - Rice paddies	-1.8300	0.4650	52	-3.9400	0.0871		-3.708	0.556	52	-6.673	<.0001
Forest fragments - Vanilla agroforests	1.1490	0.4120	52	2.7870	0.0098		-0.221	0.479	52	-0.462	0.9972
Forest fragments - Woody fallows	-1.7010	0.3790	52	-1.8500	0.0023		-1.898	0.507	52	-3.742	0.0058
Forest fragments - Herbaceous fallows	2.8080	0.4460	52	6.2950	<.0001		-0.792	0.486	52	-1.63	0.5832
Forest fragments - Rice paddies	1.4990	0.4230	52	3.5460	<.0001		-2.976	0.543	52	-5.477	<.0001
Vanilla agroforests - Woody fallows	-1.8500	0.4150	52	-4.4630	<.0001		-1.676	0.508	52	-3.303	0.0204
Vanilla agroforests - Herbaceous fallows	1.6580	0.4490	52	3.6920	0.0180		-0.571	0.488	52	-1.17	0.8489
Vanilla agroforests - Rice paddies	0.3500	0.4360	52	0.8040	0.0476		-2.755	0.542	52	-5.08	0.0001
Woody fallows - Herbaceous fallows	3.5090	0.4540	52	7.7270	<.0001		1.105	0.508	52	2.178	0.2656
Woody fallows - Rice paddies	2.2000	0.4270	52	5.1560	<.0001		-1.079	0.517	52	-2.086	0.3103
Herbaceous fallows - Rice paddies	-1.3080	0.4510	52	-2.9000	0.7013		-2.184	0.537	52	-4.068	0.0021
	Construction						Firewood				
	estimate	SE	df	t.ratio	p.value		estimate	SE	df	t.ratio	p.value
Old-growth forests - Forest fragments	-4.6294	0.493	52	-9.381	<.0001		-1.53985	0.478	52	-3.219	0.0255
Old-growth forests - Vanilla agroforests	-0.582	0.465	52	-1.253	0.8086		-1.48631	0.478	52	-3.11	0.0341
Old-growth forests - Woody fallows	-3.4442	0.466	52	-7.393	<.0001		-4.40218	0.557	52	-7.9	<.0001
Old-growth forests - Herbaceous fallows	0.0214	0.464	52	0.046	1		-0.09792	0.47	52	-0.208	0.9999
Old-growth forests - Rice paddies	0.1141	0.464	52	0.246	0.9999		-1E-06	0.47	52	0	1
Forest fragments - Vanilla agroforests	4.0474	0.473	52	8.553	<.0001		0.05354	0.467	52	0.115	1
Forest fragments - Woody fallows	1.1852	0.364	52	3.257	0.023		-2.86233	0.52	52	-5.506	<.0001
Forest fragments - Herbaceous fallows	4.6508	0.482	52	9.647	<.0001		1.441929	0.466	52	3.093	0.0356
Forest fragments - Rice paddies	4.7435	0.483	52	9.821	<.0001		1.539849	0.466	52	3.304	0.0203
Vanilla agroforests - Woody fallows	-2.8621	0.446	52	-6.41	<.0001		-2.91587	0.522	52	-5.591	<.0001
Vanilla agroforests - Herbaceous fallows	0.6034	0.452	52	1.334	0.7647		1.388389	0.466	52	2.981	0.0473
Vanilla agroforests - Rice paddies	0.6961	0.452	52	1.539	0.6409		1.486309	0.466	52	3.192	0.0274
Woody fallows - Herbaceous fallows	3.4656	0.454	52	7.638	<.0001		4.304254	0.546	52	7.883	<.0001
Woody fallows - Rice paddies	3.5583	0.454	52	7.83	<.0001		4.402174	0.547	52	8.052	<.0001
Herbaceous fallows - Rice paddies	0.0927	0.452	52	0.205	0.9999		0.09792	0.458	52	0.214	0.9999

contrast	Charcoal						Fodder				
	estimate	SE	df	t.ratio	p.value		estimate	SE	df	t.ratio	p.value
Old-growth forests - Forest fragments	-0.644	0.464	52	-1.389	0.733		0.000	0.471	52	0	1
Old-growth forests - Vanilla agroforests	-0.459	0.463	52	-0.99	0.9192		-0.199	0.472	52	-0.423	0.9982
Old-growth forests - Woody fallows	-1.140	0.464	52	-2.464	0.154		-2.360	0.49	52	-4.805	0.0002
Old-growth forests - Herbaceous fallows	0.000	0.463	52	0	1		-0.449	0.473	52	-0.949	0.9317
Old-growth forests - Rice paddies	-0.100	0.463	52	-0.217	0.9999		-2.830	0.5	52	-5.658	<.0001
Forest fragments - Vanilla agroforests	0.186	0.451	52	0.412	0.9984		-0.199	0.459	52	-0.435	0.9979
Forest fragments - Woody fallows	-0.500	0.449	52	-1.112	0.8741		-2.360	0.478	52	-4.927	0.0001
Forest fragments - Herbaceous fallows	0.644	0.451	52	1.427	0.7104		-0.449	0.46	52	-0.974	0.924
Forest fragments - Rice paddies	0.544	0.451	52	1.205	0.8323		-2.830	0.488	52	-5.795	<.0001
Vanilla agroforests - Woody fallows	-0.685	0.45	52	-1.522	0.6519		-2.160	0.478	52	-4.511	0.0005
Vanilla agroforests - Herbaceous fallows	0.459	0.451	52	1.017	0.9103		-0.249	0.461	52	-0.54	0.9942
Vanilla agroforests - Rice paddies	0.358	0.451	52	0.794	0.9673		-2.630	0.487	52	-5.391	<.0001
Woody fallows - Herbaceous fallows	1.140	0.452	52	2.531	0.1339		1.910	0.478	52	3.993	0.0027
Woody fallows - Rice paddies	1.040	0.452	52	2.311	0.2084		-0.471	0.464	52	-1.017	0.9102
Herbaceous fallows - Rice paddies	-0.100	0.451	52	-0.223	0.9999		-2.380	0.486	52	-4.889	0.0001

contrast	Weaving					
	estimate	SE	df	t.ratio	p.value	
Old-growth forests - Forest fragments	-0.882	0.467	52	-1.889	0.4203	
Old-growth forests - Vanilla agroforests	-0.456	0.466	52	-0.979	0.9227	
Old-growth forests - Woody fallows	-0.463	0.466	52	-0.994	0.9178	
Old-growth forests - Herbaceous fallows	0.000	0.465	52	0	1	
Old-growth forests - Rice paddies	-1.370	0.468	52	-2.928	0.0539	
Forest fragments - Vanilla agroforests	0.426	0.454	52	0.938	0.9348	
Forest fragments - Woody fallows	0.419	0.454	52	0.922	0.9391	
Forest fragments - Herbaceous fallows	0.882	0.454	52	1.94	0.3901	
Forest fragments - Rice paddies	-0.490	0.453	52	-1.082	0.8864	
Vanilla agroforests - Woody fallows	-0.007	0.454	52	-0.016	1	
Vanilla agroforests - Herbaceous fallows	0.456	0.453	52	1.005	0.9141	
Vanilla agroforests - Rice paddies	-0.916	0.455	52	-2.014	0.3488	
Woody fallows - Herbaceous fallows	0.463	0.453	52	1.021	0.9087	
Woody fallows - Rice paddies	-0.908	0.455	52	-1.998	0.3575	
Herbaceous fallows - Rice paddies	-1.370	0.456	52	-3.008	0.0442	

Table S9. Mean number of species used per village with standard errors across land-use types in north-eastern Madagascar

Land-use types	Mean	SE
Old-growth forests	0.2	0.2
Forest fragments	25.6	2.8
Vanilla agroforests	19.3	4.3
Woody fallows	49.2	6.2
Herbaceous fallows	5.7	2.1
Rice paddies	15.5	1.4

Table S10. Multiple comparisons of land-use types in terms of the number of species used by households per village.

Contrast	estimate	SE	df	t.ratio	p.value
Old-growth forests - Forest fragments	-4.6315	1.01	53	-4.587	0.0004
Old-growth forests - Vanilla agroforests	-4.1837	1.016	53	-4.12	0.0018
Old-growth forests - Woody fallows	-5.2498	1.006	53	-5.217	<.0001
Old-growth forests - Herbaceous fallows	-2.9839	1.034	53	-2.885	0.0596
Old-growth forests - Rice paddies	-4.2255	1.012	53	-4.177	0.0015
Forest fragments - Vanilla agroforests	0.4478	0.221	53	2.022	0.3439
Forest fragments - Woody fallows	-0.6184	0.172	53	-3.594	0.0088
Forest fragments - Herbaceous fallows	1.6476	0.309	53	5.326	<.0001
Forest fragments - Rice paddies	0.406	0.212	53	1.919	0.4022
Vanilla agroforests - Woody fallows	-1.0662	0.202	53	-5.276	<.0001
Vanilla agroforests - Herbaceous fallows	1.1998	0.328	53	3.662	0.0072
Vanilla agroforests - Rice paddies	-0.0418	0.237	53	-0.176	1
Woody fallows - Herbaceous fallows	2.2659	0.297	53	7.625	<.0001
Woody fallows - Rice paddies	1.0244	0.192	53	5.332	<.0001
Herbaceous fallows - Rice paddies	-1.2416	0.318	53	-3.902	0.0035

Table S11. Mean of the percentage of species used from each land-use type in north-eastern Madagascar based on their origin (the endemic, native and exotic) and growth forms (trees, shrubs and lianas, herbs)

Land-use types	Species origin	Mean percentage ± SE		Land-use types	Growth forms	Mean percentage ± SE
Old-growth forests	Endemics	5 ±5		Old-growth forests	Herb	0 ±0
	Natives	0 ±0			Shrub & Liana	0 ±0
	Exotics	0 ±0			Tree	10 ±10
Forest fragments	Endemics	42.9 ±3.1		Forest fragments	Herb	10.5 ±2.2
	Natives	19.4 ±2.6			Shrub & Liana	6.4 ±1.6
	Exotics	11 ±2.8			Tree	83.2 ±3
Vanilla agroforests	Endemics	27 ±6.2		Vanilla agroforests	Herb	34.8 ±3.1
	Natives	31.1 ±4.3			Shrub & Liana	8.8 ±2
	Exotics	30.1 ±4.3			Tree	56.4 ±2
Woody fallows	Endemics	19.4 ±1.7		Woody fallows	Herb	41.6 ±2
	Natives	37.1 ±2			Shrub & Liana	9.1 ±1.3
	Exotics	32.7 ±1.8			Tree	49.3 ±2.5
Herbaceous fallows	Endemics	63.7 ±9.9		Herbaceous fallows	Herb	78.7 ±10.5
	Natives	2 ±2			Shrub & Liana	3.2 ±2.1
	Exotics	24.3 ±7.1			Tree	8.1 ±5.3
Rice paddies	Endemics	5.5 ±2		Rice paddies	Herb	91.2 ±3.3
	Natives	46.9 ±3.6			Shrub & Liana	2.2 ±1.2
	Exotics	42.6 ±2.4			Tree	6.6 ±2.4

Figures

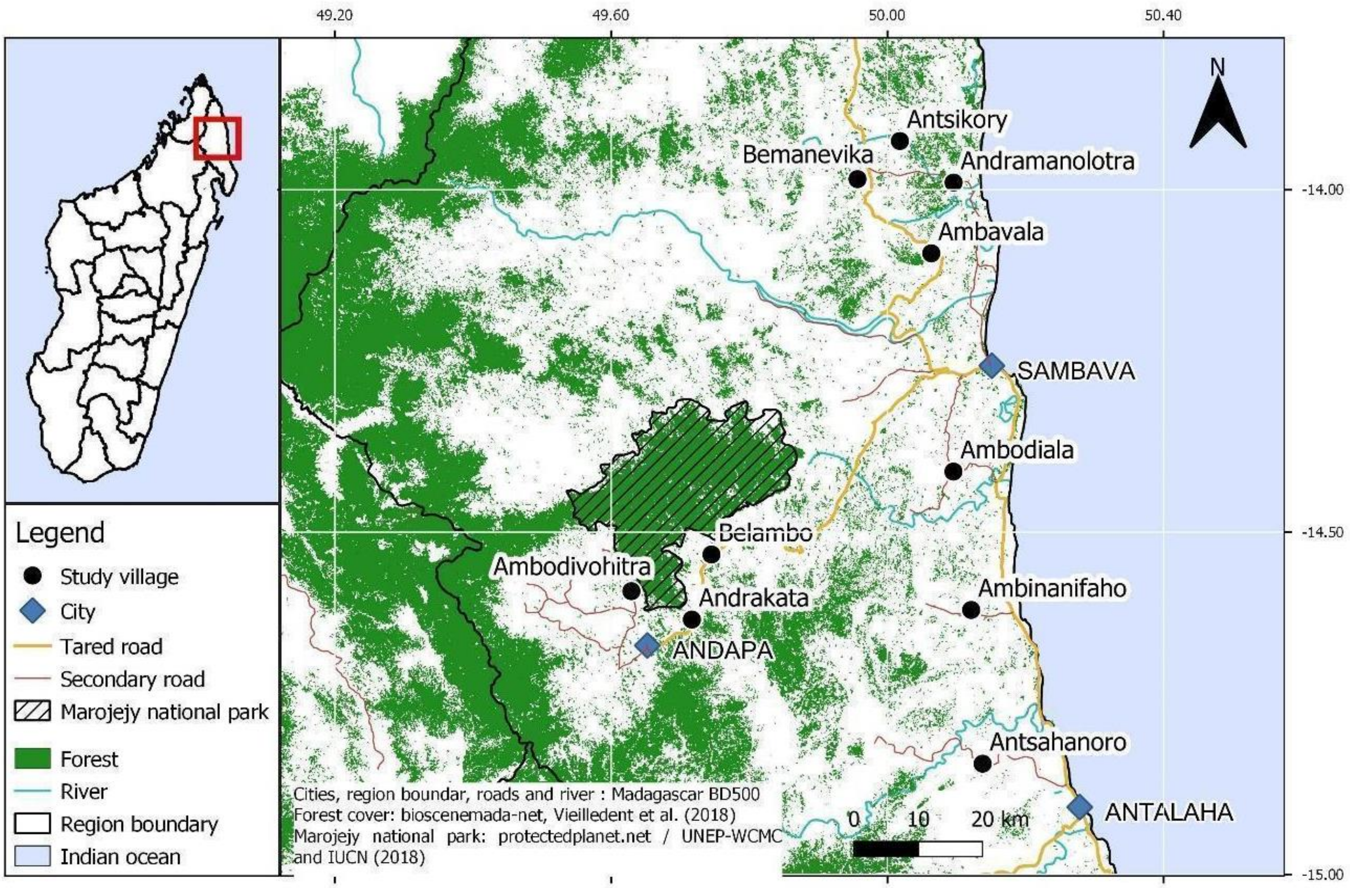

Figure S1. Map of study villages with the forest cover of the study region in 2017 from (Vieilledent et al. 2018)

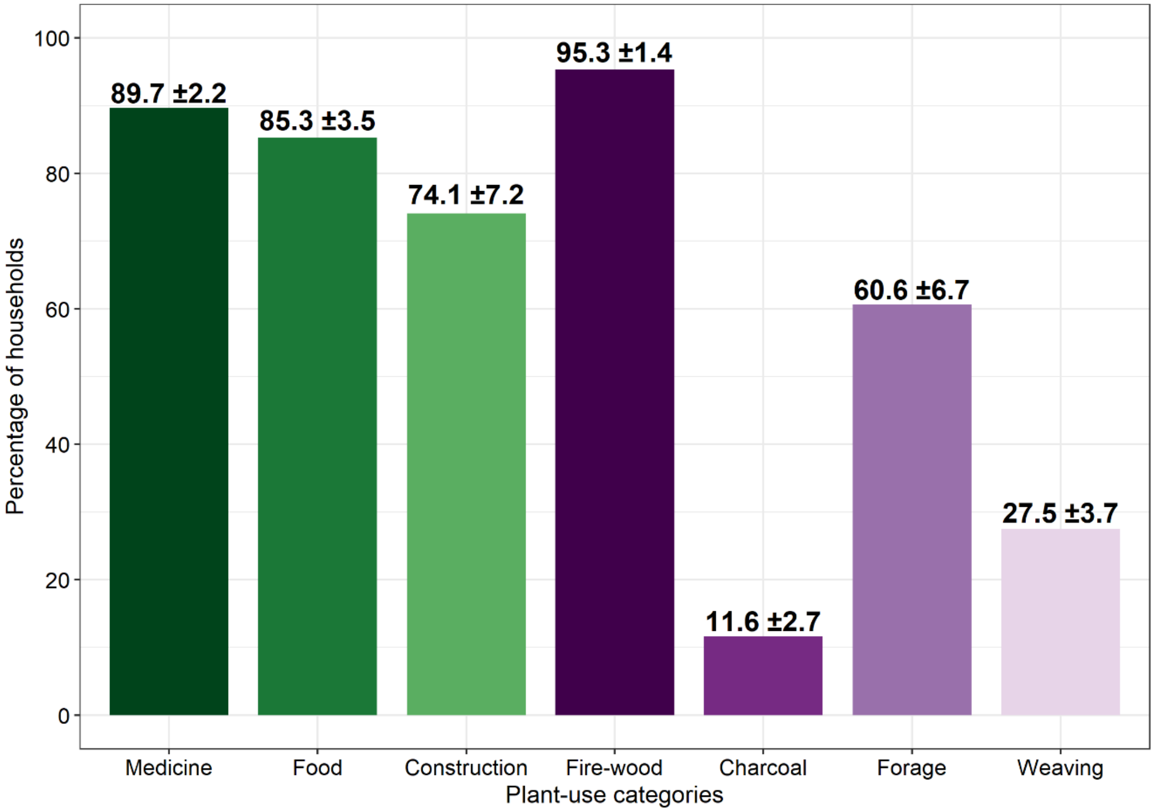

Figure S2. Mean percentage and standard error of households using species for each category of use in north-eastern Madagascar

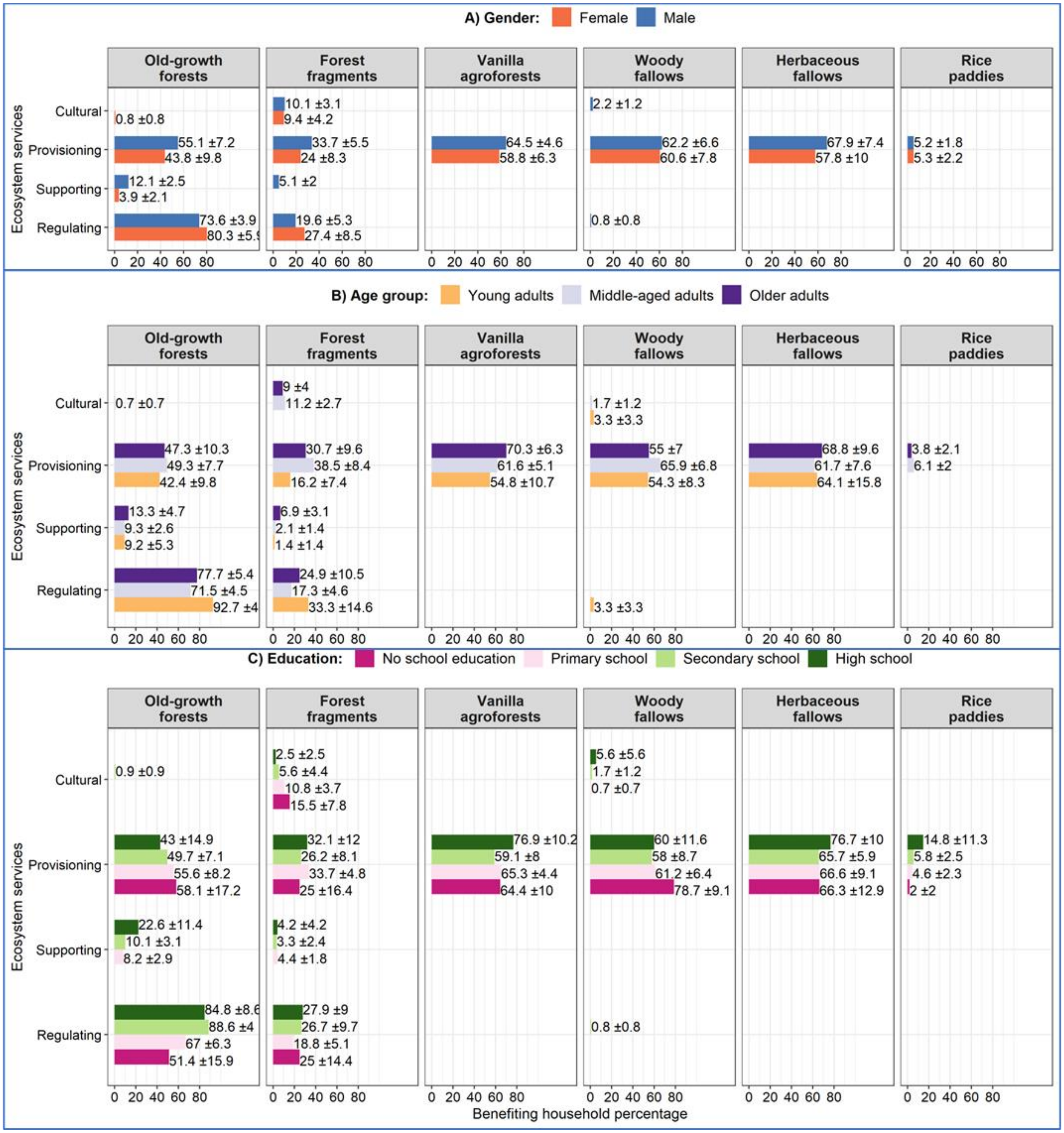

Figure S3. Characteristics of the households relying on different land uses for ecosystem services (excluding crop yields) based on gender (a), age group (b) and education level (c) of household's heads. Number on a bar represents the mean percentage (±SE) of households belonging to the group (gender, age group pr education) with access to the land use type reporting the corresponding benefits, i.e. supporting, regulating, provisioning or cultural ecosystem services for each land use type.

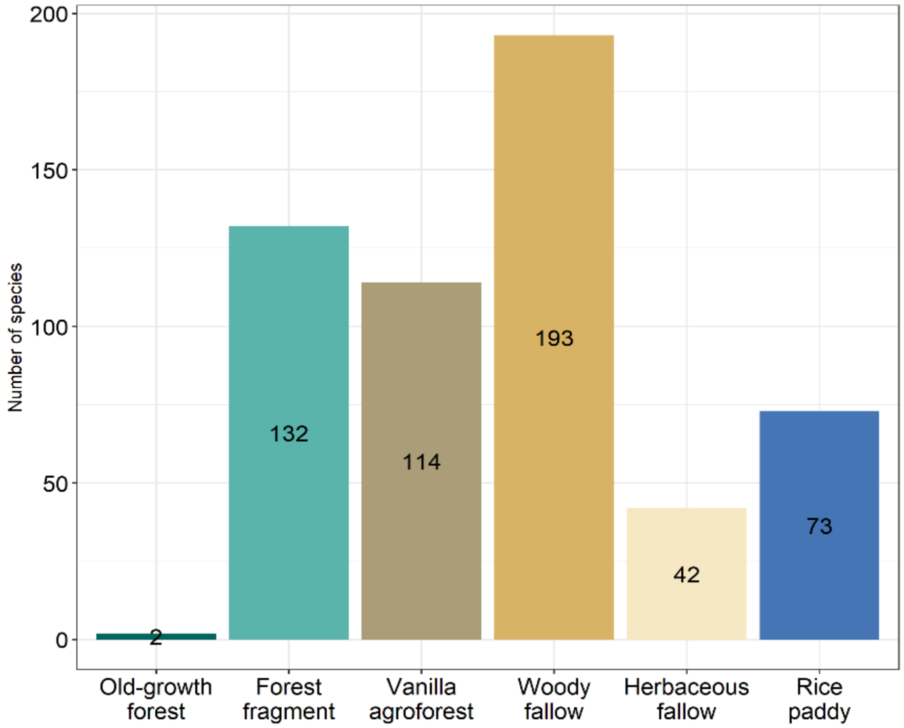

Figure S4. Total number of species used across land-use types in north-eastern Madagascar

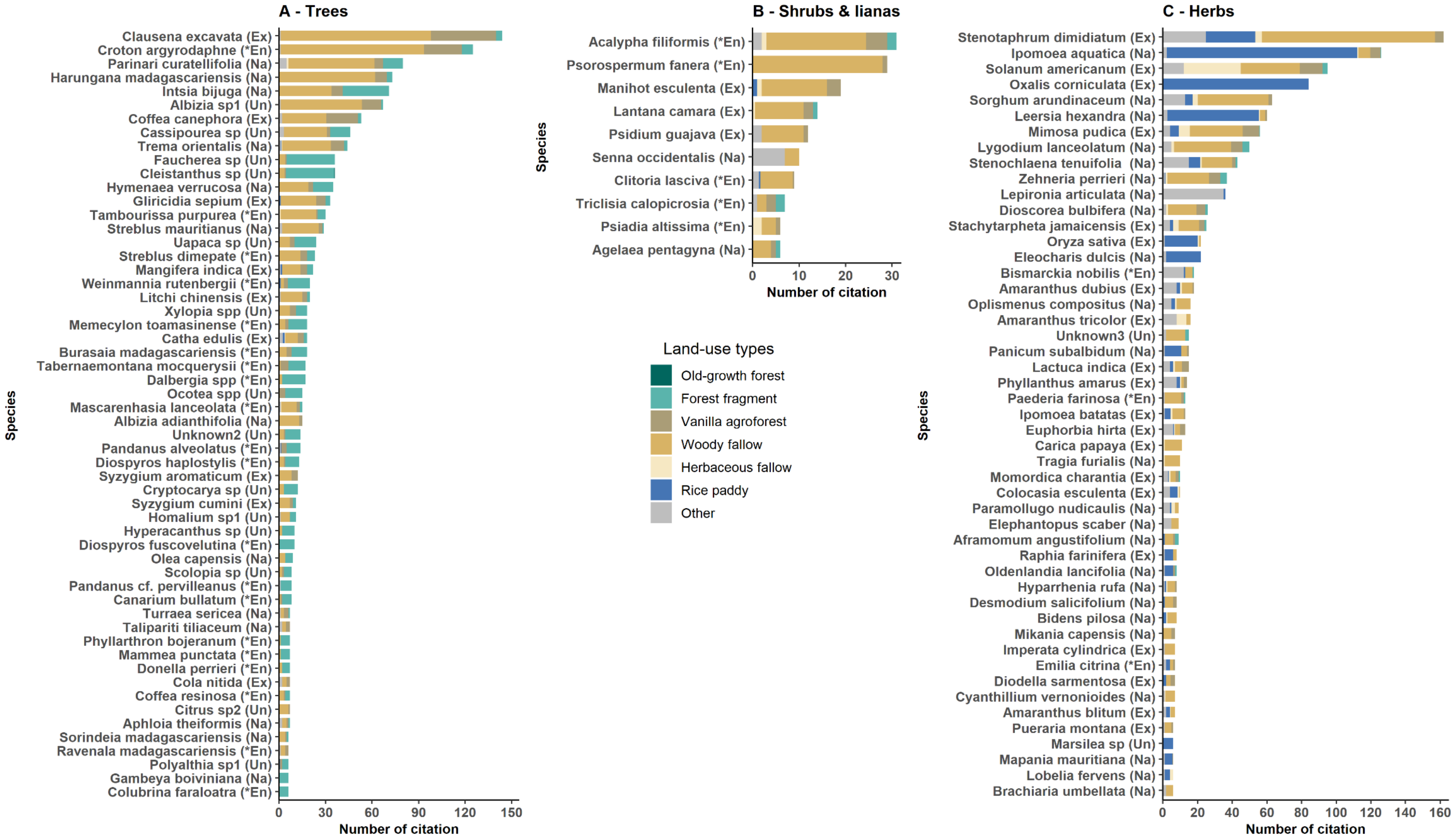

Figure S5. Location to collect plant species cited by at least 6 households and the number of their citation across the study region. One bar represents one species and the length of the bar represents the number of citations by households across the 10 villages. Species names with "*En" are endemic species, "Na" for native, "Ex" for exotic and "Un" for unknown origin

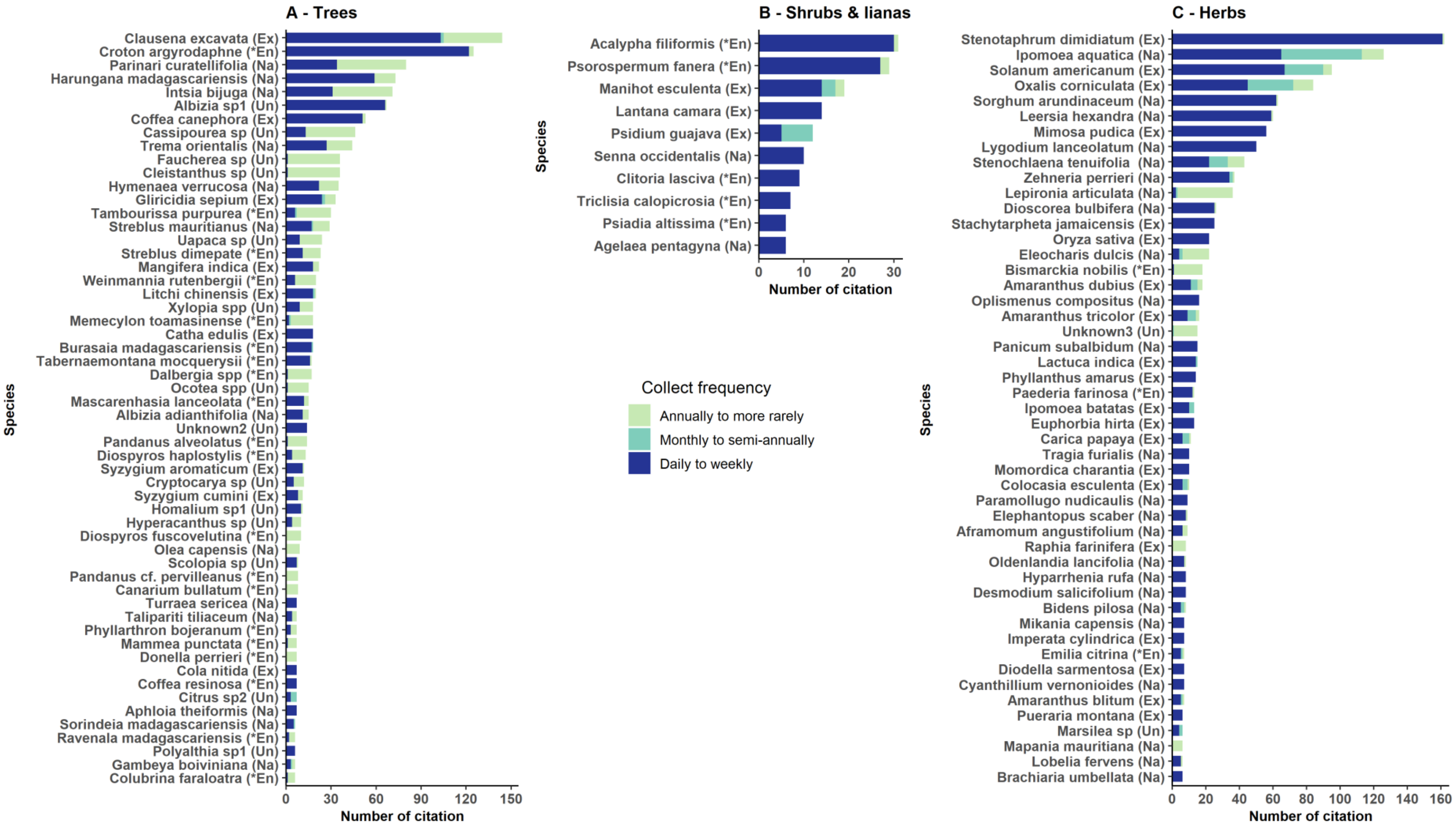

Figure S6. Collect frequency of plant species cited by at least 6 households and the number of their citations across the study region. One bar represents one species and the length of the bar represents the number of citations by households across the 10 villages. Species names with "*En" are endemic species, "Na" for native, "Ex" for exotic and "Un" for unknown origin

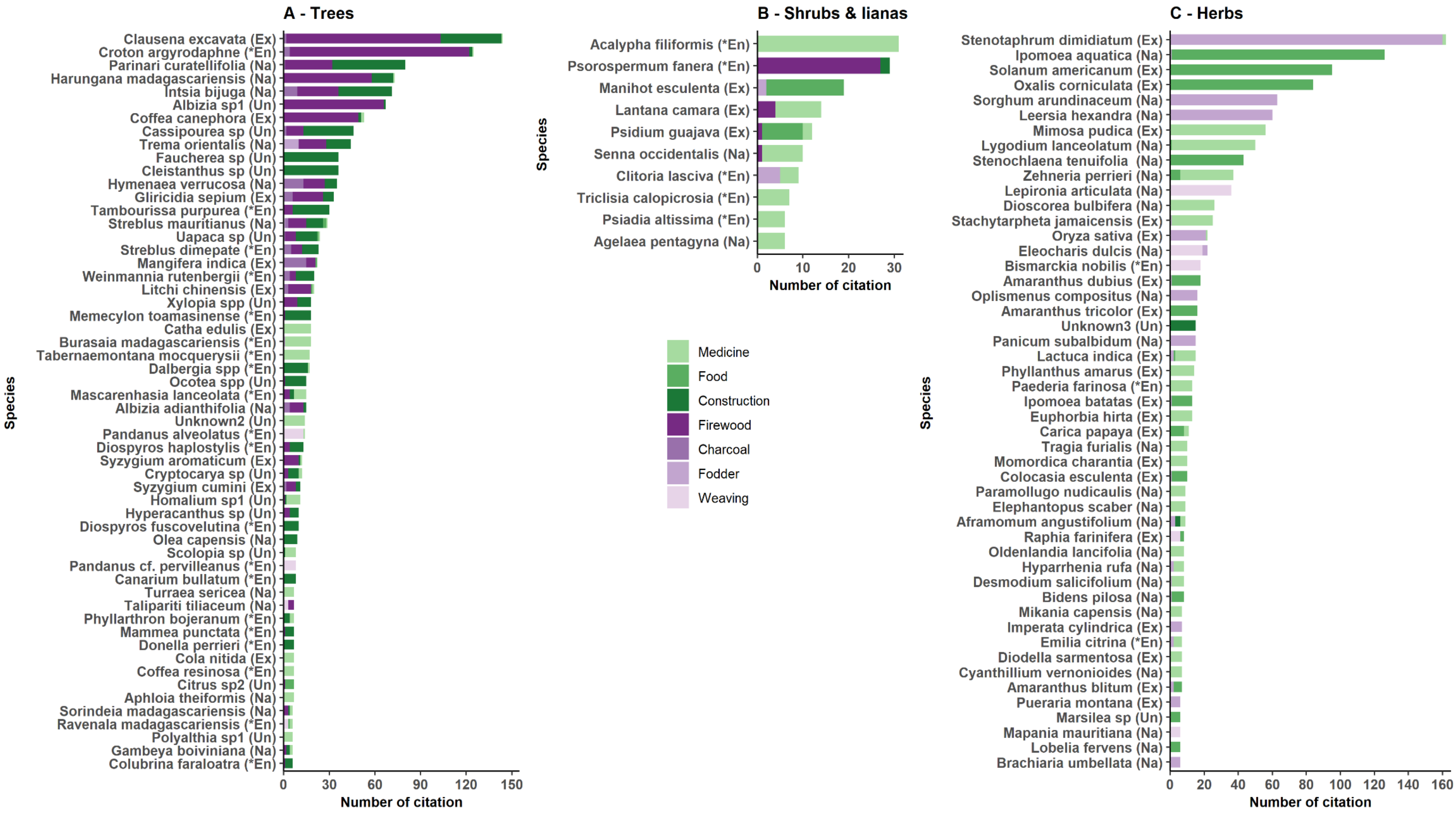

Figure S7. Different use of plant species cited by at least 6 households and the number of their citations across the study region. One bar represents one species and the length of the bar represents the number of citations by households across the 10 villages. Species names with "*En" are endemic species, "Na" for native, "Ex" for exotic and "Un" for unknown origin
